# Supplementary material for: Epigenetic alterations affecting hematopoietic regulatory networks as drivers of mixed myeloid/lymphoid leukemia
Source: Nat Commun. 2024 Jul 7;15:5693. doi: 10.1038/s41467-024-49811-y (PMC11228033; doi:10.1038/s41467-024-49811-y)
Supplement: Supplementary file 1 — Supplementary Information [file 41467_2024_49811_MOESM1_ESM.pdf]

SUPPLEMENTARY INFORMATION

Epigenetic alterations affecting hematopoietic regulatory networks as drivers of mixed myeloid/lymphoid leukemia

Roger Mulet-Lazaro[1,2], Stanley van Herk[1,2], Margit Nuetzel[3], Aniko Sijs-Szabo[1], Noelia Díaz[4,5], Katherine Kelly[6], Claudia Erpelinck-Verschueren[1,2], Lucia Schwarzfischer-Pfeilschifter[3], Hanna Stanewsky[3], Ute Ackermann[3], Dagmar Glatz[3], Johanna Raithel[3], Alexander Fischer[3], Sandra Pohl[3,7], Anita Rijneveld[1], Juan M Vaquerizas[4,8,9], Christian Thiede[10], Christoph Plass[6], Bas J. Wouters[1,2,*], Ruud Delwel[1,2, *], Michael Rehli[3,11,*], Claudia Gebhard[3,11,*]

[1] Department of Hematology, Erasmus MC Cancer Institute, Rotterdam, the Netherlands
[2] Oncode Institute, Utrecht, the Netherlands
[3] Department of Internal Medicine III, University Hospital Regensburg, Regensburg, Germany
[4] Max Planck Institute for Molecular Biomedicine, Muenster, Germany
[5] Renewable Marine Resources Department, Institute of Marine Sciences (ICM-CSIC), Barcelona, Spain
[6] Division of Cancer Epigenomics, German Cancer Research Center (DKFZ), Heidelberg, Germany
[7] Department of Conservative Dentistry and Periodontology, University Hospital Regensburg, Regensburg, Germany
[8] MRC London Institute of Medical Sciences, London, United Kingdom
[9] Institute of Clinical Sciences, Faculty of Medicine, Imperial College London, Hammersmith Hospital 8 Campus, London, United Kingdom
[10] Medizinische Klinik und Poliklinik I, Universitätsklinikum Carl Gustav Carus, Dresden, Germany
[11] Leibniz Institute for Immunotherapy (LIT), Regensburg, Germany

* These authors contributed equally to this work.

Corresponding authors: Ruud Delwel (h.delwel@erasmusmc.nl), Michael Rehli (michael.rehli@klinik.uni-regensburg.de), Claudia Gebhard (claudia.gebhard@klinik.uni-regensburg.de), Bas J. Wouters (b.wouters@erasmusmc.nl)

SUPPLEMENTARY FIGURES 2
SUPPLEMENTARY RESULTS 24
The mutational landscape of CIMPs is similar to that of ETP-ALL 24
Analysis of motif activity inferred from ATAC-seq pinpoints key dysregulated TFs involved in lineage specification 24
Supervised analyses of ATAC-seq and ChIP-seq data identify epigenetic signatures in line with gene expression patterns 25
Additional examples of altered 3D genome structure 26
Loss of *CEBPA* plays a critical role in shaping the leukemic epigenome 27
SUPPLEMENTARY REFERENCES 28

SUPPLEMENTARY FIGURES

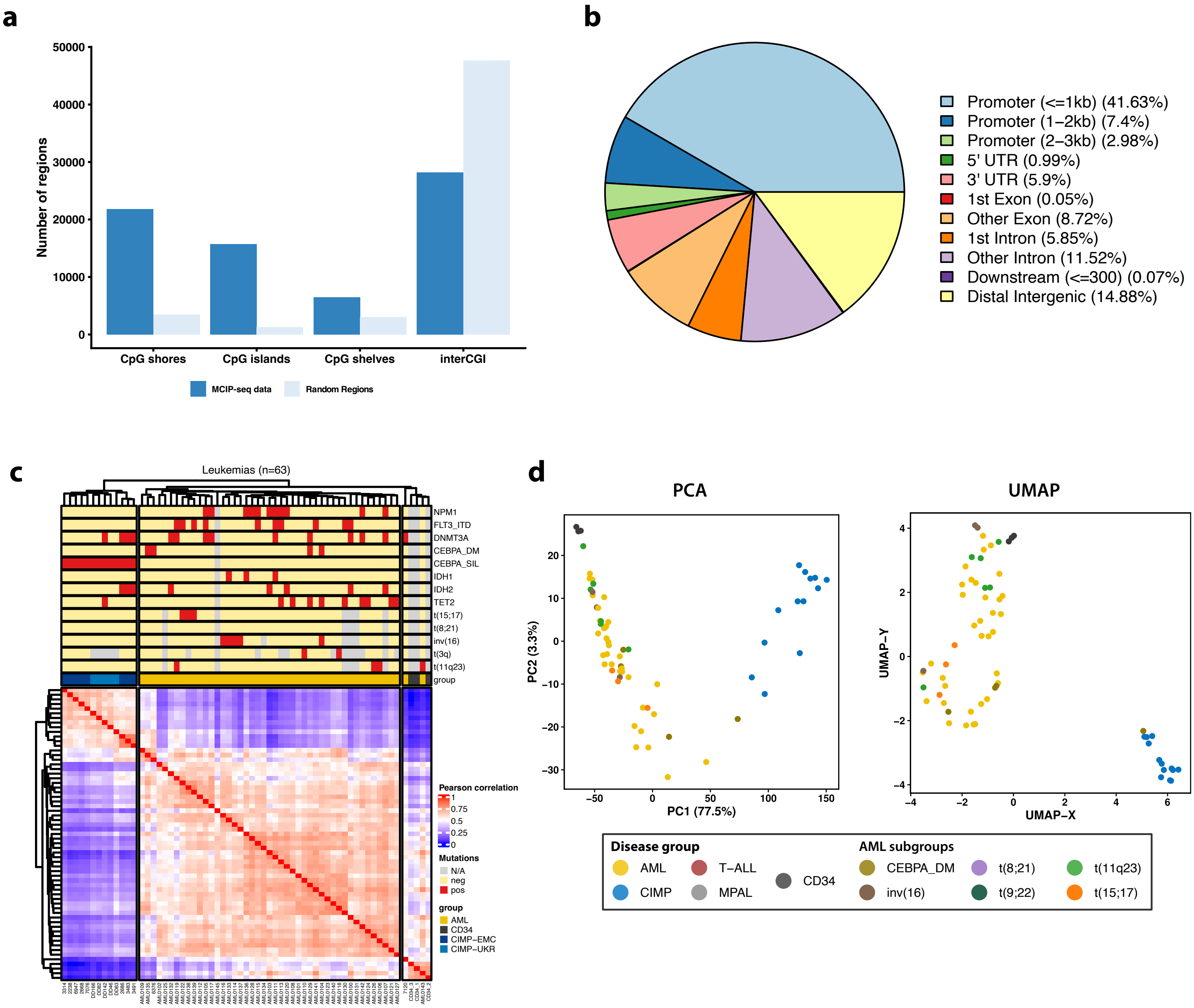

Supplementary Figure 1. MCIP-seq data analysis. a Coverage of CpG islands, shores, shelves and inter-CGI by MCIP-seq data, compared to an equal set of randomly selected regions. The plot shows enrichment at CpG-rich regions and depletion at inter-CGI regions relative to the random set. **b** Functional annotation of methylated regions detected by MCIP-seq. **c** Pearson correlation heatmap of MCIP-seq data from AML (n=50) and CIMP (n=13) cases. **d** MCIP-seq data dimensionality reduction separates CIMP from AML and CD34+ HSPCs using Principal Component Analysis (PCA, left) and Uniform Manifold Approximation and Projection (UMAP, right). Relevant AML subgroups known to exhibit distinct patterns of gene expression are highlighted: inv(16), t(8;21), t(9;22), t(15;17), 11q23 (MLL) rearrangements [t(11q23)], CEBPA double mutations (CEBPA_DM).

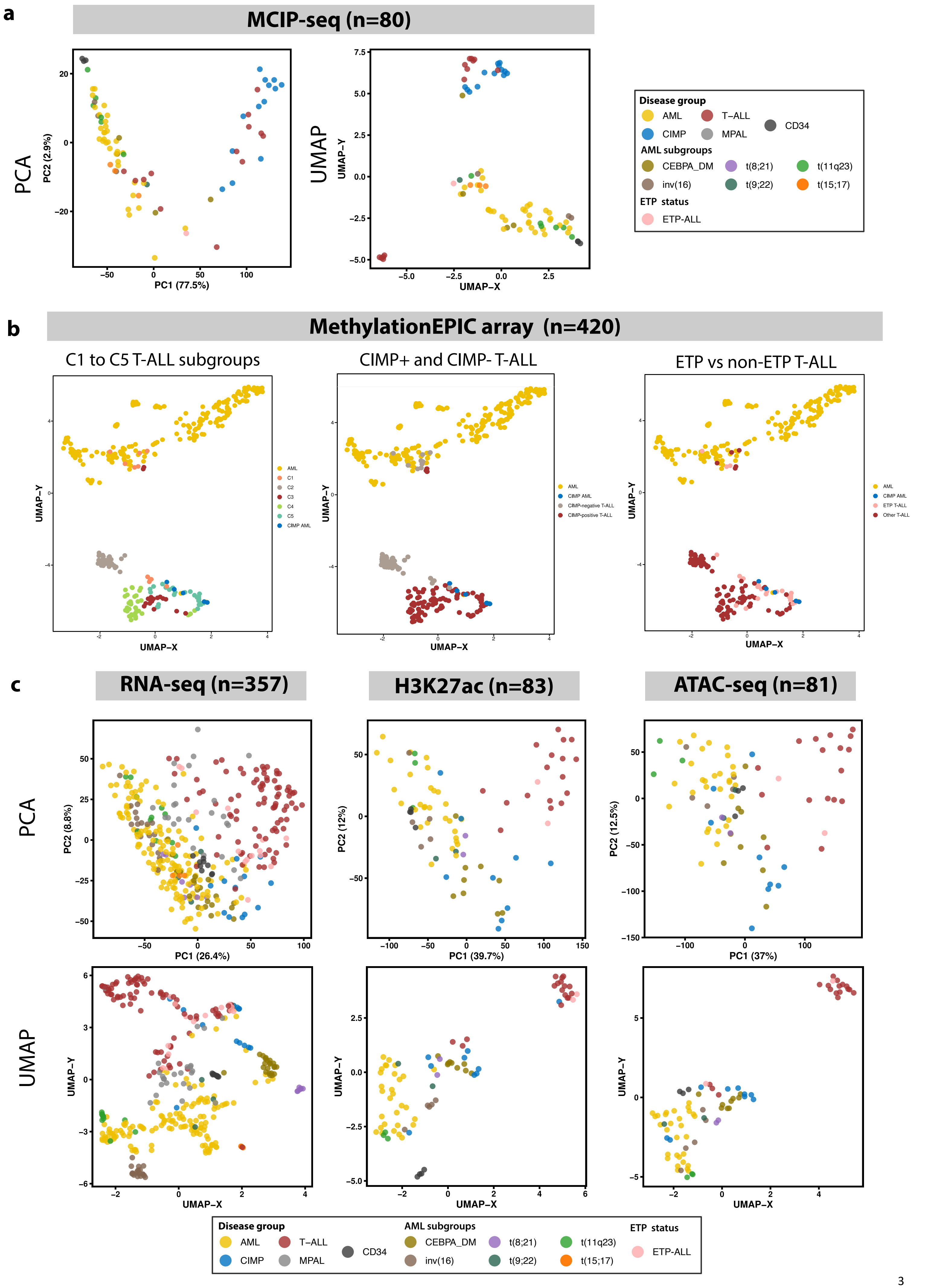
a
MCIP-seq (n=80)
PCA
UMAP
PC1 (77.5%)
PC2 (2.9%)
UMAP–X
UMAP–Y
Disease group
AML
T–ALL
CD34
CIMP
MPAL
AML subgroups
CEBPA_DM
t(8;21)
t(11q23)
inv(16)
t(9;22)
t(15;17)
ETP status
ETP-ALL
b
MethylationEPIC array (n=420)
C1 to C5 T-ALL subgroups
CIMP+ and CIMP- T-ALL
ETP vs non-ETP T-ALL
AML
C1
C2
C3
C4
C5
CIMP AML
CIMP-negative T-ALL
CIMP-positive T-ALL
ETP T-ALL
Other T-ALL
c
RNA-seq (n=357)
H3K27ac (n=83)
ATAC-seq (n=81)
PC1 (26.4%)
PC2 (8.8%)
PC1 (39.7%)
PC2 (12%)
PC1 (37%)
PC2 (12.5%)

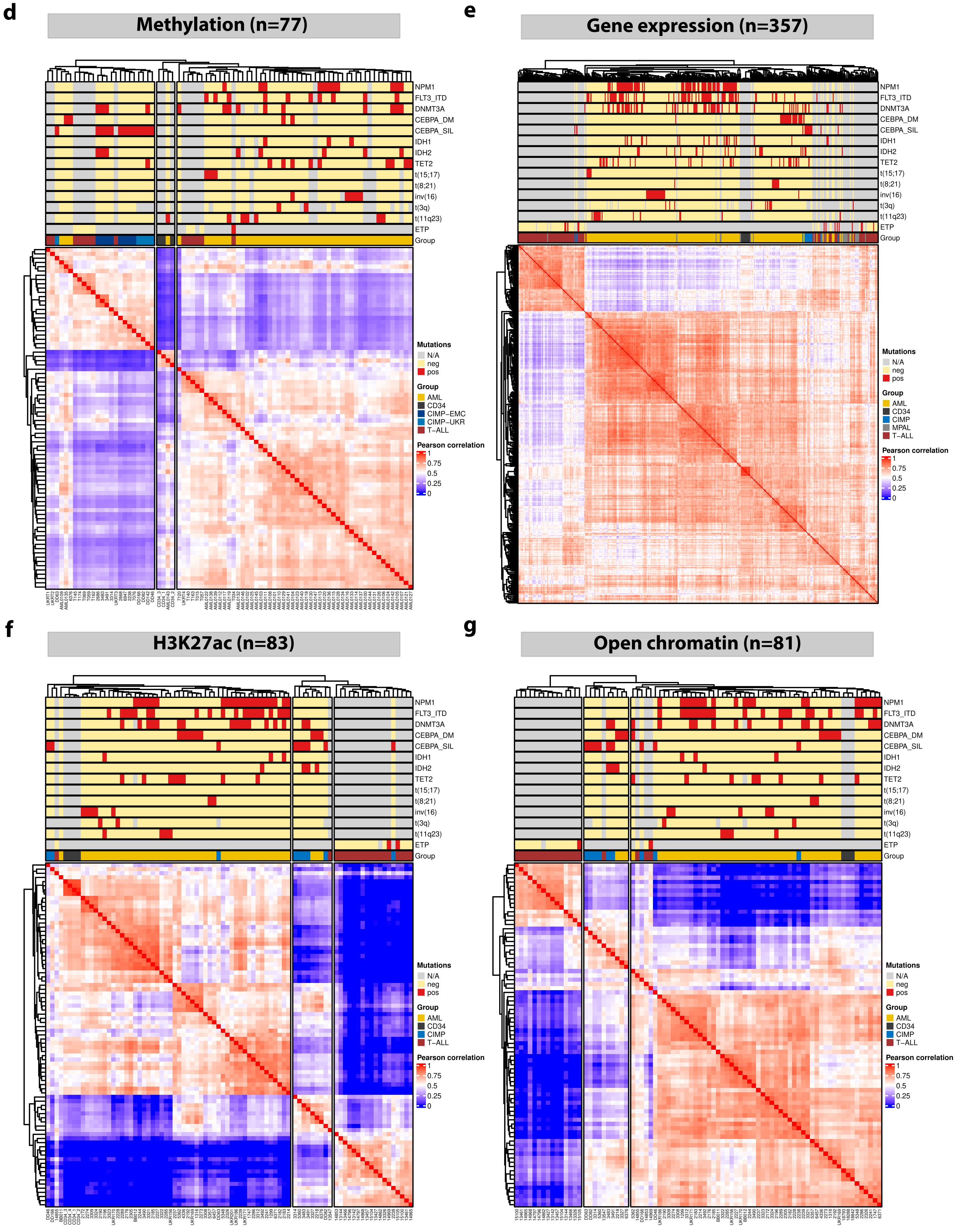
d
Methylation (n=77)
e
Gene expression (n=357)
f
H3K27ac (n=83)
g
Open chromatin (n=81)
NPM1
FLT3_ITD
DNMT3A
CEBPA_DM
CEBPA_SIL
IDH1
IDH2
TET2
t(15;17)
t(8;21)
inv(16)
t(3q)
t(11q23)
ETP
Group
Mutations
N/A
neg
pos
Group
AML
CD34
CIMP–EMC
CIMP–UKR
CIMP
MPAL
T–ALL
Pearson correlation
1
0.75
0.5
0.25
0

Supplementary Figure 2. Epigenetic and transcriptional landscape of CIMP, AML, T-ALL and CD34+ cells. a Dimensionality reduction with either PCA (left) or UMAP (right) of MCIP-seq data, comparing AML (n=50), CIMP (n=13) and T-ALL (n=14). Relevant AML subgroups known to exhibit distinct patterns of gene expression are colored: inv(16), t(8;21), t(9;22), t(15;17), 11q23 (MLL) rearrangements [t(11q23)], CEBPA double mutations (CEBPA_DM). ETP-ALL cases identified on the basis of their immunophenotype are also labelled. **b** UMAP dimensionality reduction of MethylationEPIC array data from CIMP leukemias (n=5) in combination with previously published AML (n=272) and T-ALL (n=143) data. The T-ALL samples are labelled as follows: DNA methylation-driven clusters identified by Touzart et al. [1], where C1 has the lowest methylation and C5 the highest (left); CIMP-positive or CIMP-negative, also based on global methylation levels (middle); ETP or non-ETP immunophenotype (right). **c** Dimensionality reduction with either PCA or UMAP of various types of epigenomics data in AML, CIMP, T-ALL and CD34+ HSPCs. Relevant AML subgroups known to exhibit distinct patterns of gene expression are colored (see above), as well as ETP-ALL cases. **d-g**. Pearson correlation heatmaps of the data shown in **c**, with dendrograms indicating hierarchical clustering. Column names contain the patient identifier, except in the gene expression heatmap (**e**), where the high number of cases would compromise legibility.

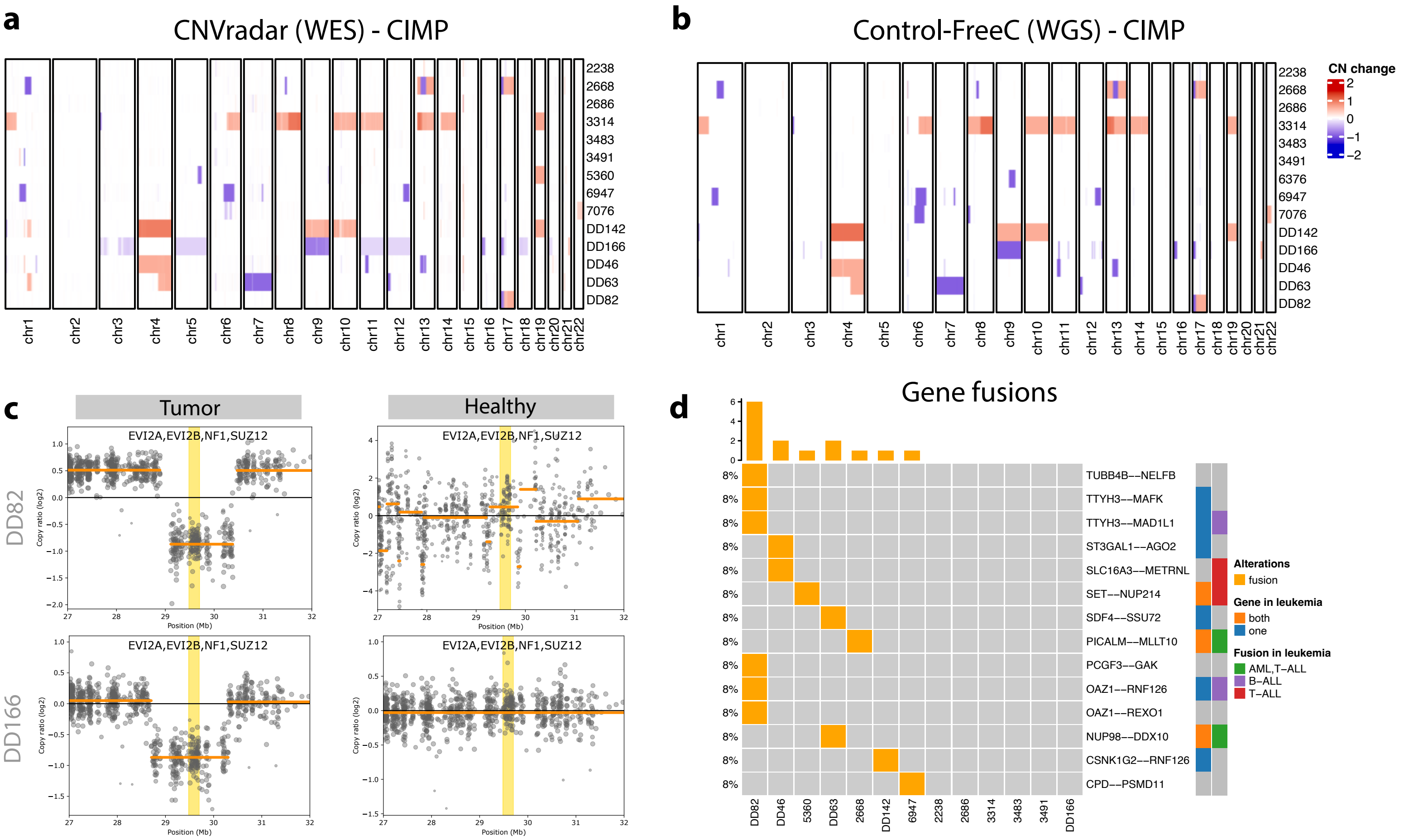

Supplementary Figure 3. Copy number and fusion gene analysis. Copy number alterations (CNAs) detected by different algorithms are represented as a heatmap where red indicates a copy number gain and blue a copy number loss. **a** Reanalysis of WES data by CNV Radar (n=14). **b** Validation of results from WES data in input DNA-sequencing with Control-FREEC (n=14). **c** Scatter plot showing CNVkit log2 copy number ratios (grey dots) and segmentation calls (orange lines) in the NF1 locus for two CIMP cases where both tumor and healthy samples were available. Copy number ratios are calculated as the depth of coverage in tumors relative to controls, following normalization. Segmentation calls group together genomic positions that are likely have the same absolute copy number based on their copy number ratios. **d** Oncoprint displaying fusion genes detected by at least 3 different software tools and not commonly found in healthy individuals. Annotations indicate which fusion genes have been reported in different leukemia sequencing projects or fusion genes whose interacting partners are involved in leukemia.

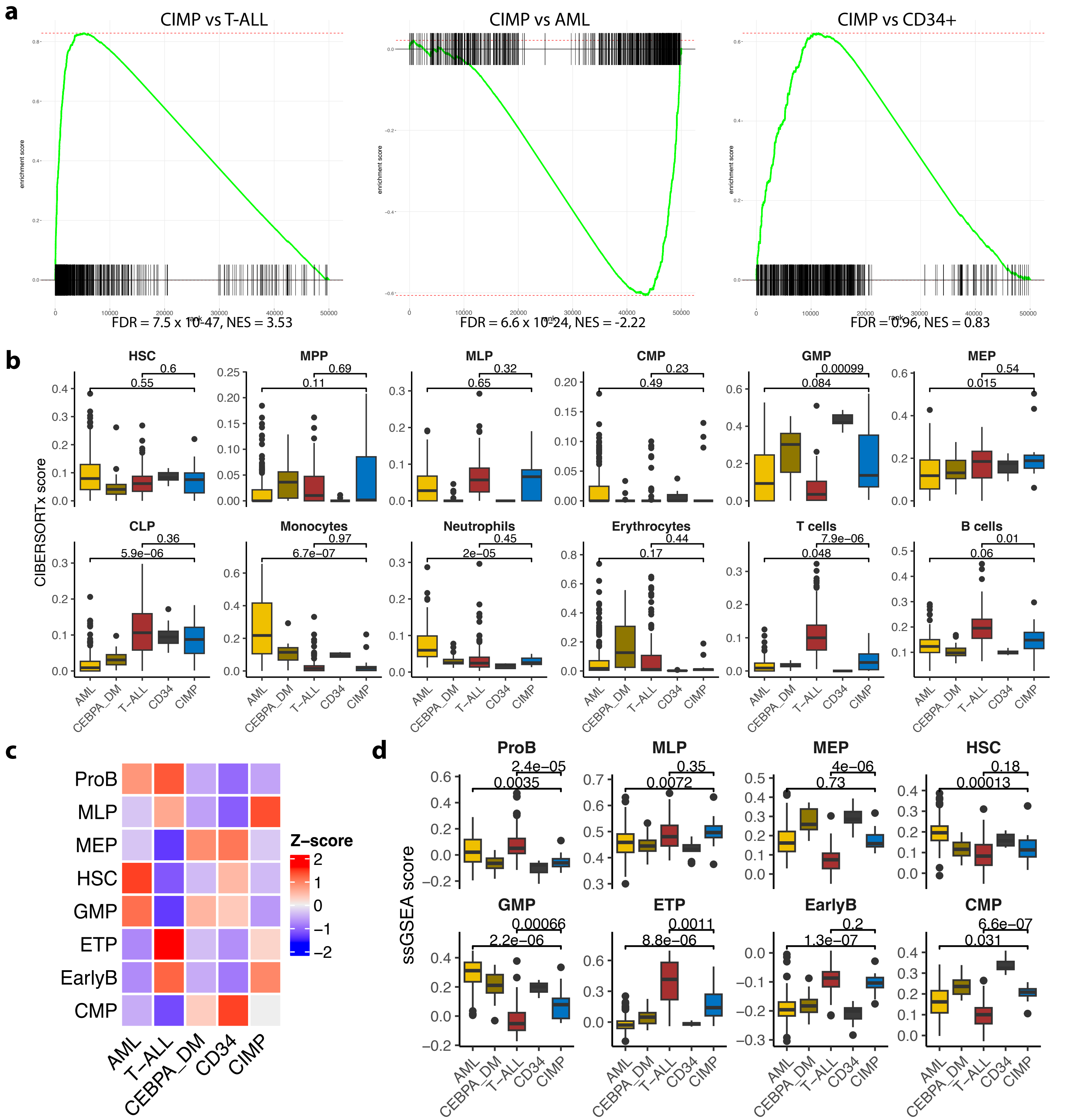

Supplementary Figure 4. Analysis of transcriptional signatures suggests that CIMPs have an early cell of origin. a GSEA enrichment plot for the gene set Zhang ETP-ALL [2] in comparisons between CIMP and T-ALL (left), AML (center) and CD34+ cells (right). Nominal p-values were calculated by a permutation test (n=10000) and adjusted for multiple comparisons with the Benjamini–Hochberg procedure. The false discovery rate (FDR) and the normalized enrichment score (NES) are indicated underneath. **b** Box plot displaying the cellular composition inferred by CIBERSORTx for each leukemia subgroup (or CD34+ cells) based on a signature derived from the Atlas of Human Blood Cells [3] (same dataset as in **Figure 2f**). The analysis was performed on a mixture matrix containing RNA-seq raw counts from multiple leukemia subgroups. The lower and upper edges of the boxplots represent the first and third quartiles, respectively; the horizontal line inside the box indicates the median. The whiskers extend to the most extreme values within the range between the median and 1.5 times the interquartile range. Lines between boxes show the p-value from a two-sided Wilcoxon test. **c** Heatmap displaying scores of single sample GSEA (ssGSEA) analysis with gene sets derived from various hematopoietic fractions by the Dick group [4]. The analysis was conducted on fragments per million (FPM)-normalized RNA-seq data from multiple leukemia subgroups, followed by row-wise Z-score normalization. **d** Box plot showing the ssGSEA results from **c**. See **b** for a description of the box plot components and the statistical methodology. In both CIBERSORT and ssGSEA, scores were calculated for every sample and aggregated by disease groups: CIMP (n=13), AML (n=189), CEBPA double mutant (DM) AML (n=22), T-ALL (n=100). The CEBPA DM subgroup was analyzed separately from other AMLs owing to its similarity with CIMP leukemias.

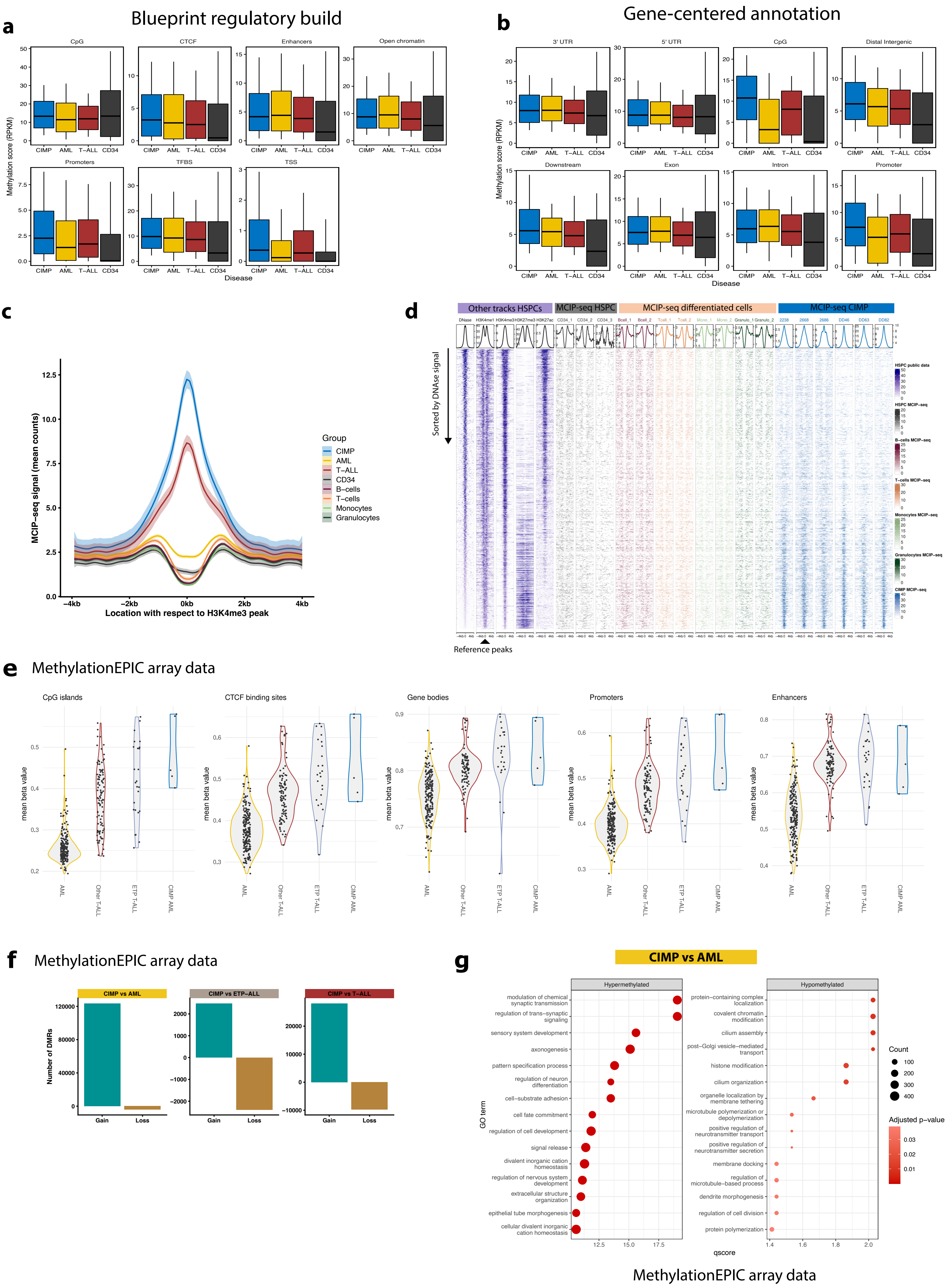
a
Blueprint regulatory build
CpG
CTCF
Enhancers
Open chromatin
Promoters
TFBS
TSS
Methylation score (RPKM)
CIMP
AML
T–ALL
CD34
Disease
b
Gene-centered annotation
3' UTR
5' UTR
CpG
Distal Intergenic
Downstream
Exon
Intron
Promoter
Methylation score (RPKM)
Disease
c
MCIP–seq signal (mean counts)
Location with respect to H3K4me3 peak
Group
CIMP
AML
T–ALL
CD34
B–cells
T–cells
Monocytes
Granulocytes
d
Other tracks HSPCs
MCIP-seq HSPC
MCIP-seq differentiated cells
MCIP-seq CIMP
Sorted by DNAse signal
Reference peaks
e
MethylationEPIC array data
CpG islands
CTCF binding sites
Gene bodies
Promoters
Enhancers
mean beta value
AML
Other T-ALL
ETP T-ALL
CIMP AML
f
MethylationEPIC array data
CIMP vs AML
CIMP vs ETP–ALL
CIMP vs T–ALL
Number of DMRs
Gain
Loss
g
CIMP vs AML
Hypermethylated
Hypomethylated
GO term
qscore
Count
Adjusted p–value
modulation of chemical synaptic transmission
regulation of trans–synaptic signaling
sensory system development
axonogenesis
pattern specification process
regulation of neuron differentiation
cell–substrate adhesion
cell fate commitment
regulation of cell development
signal release
divalent inorganic cation homeostasis
regulation of nervous system development
extracellular structure organization
epithelial tube morphogenesis
cellular divalent inorganic cation homeostasis
protein–containing complex localization
covalent chromatin modification
cilium assembly
post–Golgi vesicle–mediated transport
histone modification
cilium organization
organelle localization by membrane tethering
microtubule polymerization or depolymerization
positive regulation of neurotransmitter transport
positive regulation of neurotransmitter secretion
membrane docking
regulation of microtubule–based process
dendrite morphogenesis
regulation of cell division
protein polymerization
MethylationEPIC array data

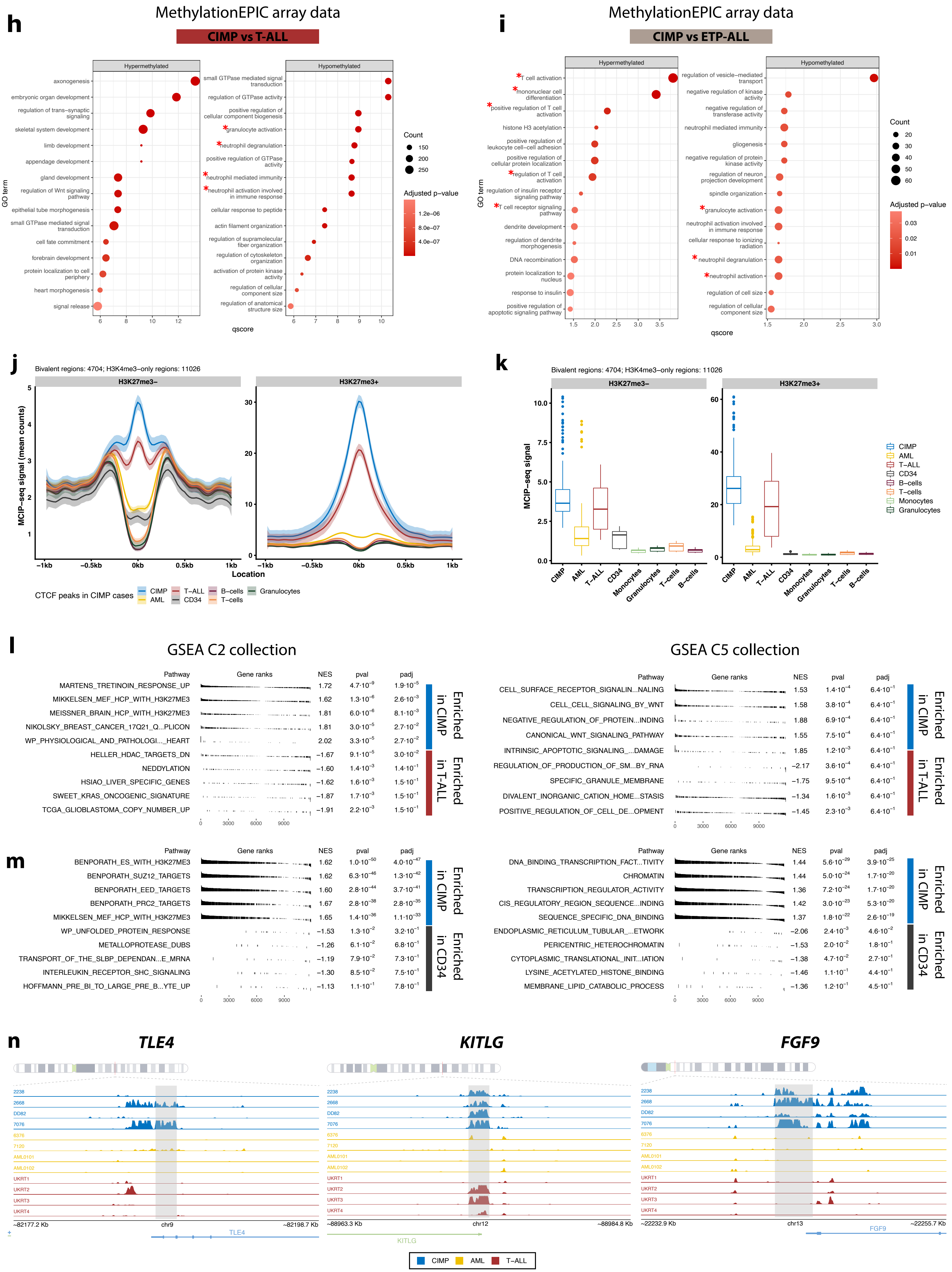
h
MethylationEPIC array data
CIMP vs T-ALL
Hypermethylated
axonogenesis
embryonic organ development
regulation of trans−synaptic signaling
skeletal system development
limb development
appendage development
gland development
regulation of Wnt signaling pathway
epithelial tube morphogenesis
small GTPase mediated signal transduction
cell fate commitment
forebrain development
protein localization to cell periphery
heart morphogenesis
signal release
Hypomethylated
small GTPase mediated signal transduction
regulation of GTPase activity
positive regulation of cellular component biogenesis
*granulocyte activation
*neutrophil degranulation
positive regulation of GTPase activity
*neutrophil mediated immunity
*neutrophil activation involved in immune response
cellular response to peptide
actin filament organization
regulation of supramolecular fiber organization
regulation of cytoskeleton organization
activation of protein kinase activity
regulation of cellular component size
regulation of anatomical structure size
GO term
qscore
Count
150
200
250
Adjusted p−value
1.2e−06
8.0e−07
4.0e−07
i
MethylationEPIC array data
CIMP vs ETP-ALL
Hypermethylated
*T cell activation
*mononuclear cell differentiation
*positive regulation of T cell activation
histone H3 acetylation
positive regulation of leukocyte cell−cell adhesion
positive regulation of cellular protein localization
*regulation of T cell activation
regulation of insulin receptor signaling pathway
*T cell receptor signaling pathway
dendrite development
regulation of dendrite morphogenesis
DNA recombination
protein localization to nucleus
response to insulin
positive regulation of apoptotic signaling pathway
Hypomethylated
regulation of vesicle−mediated transport
negative regulation of kinase activity
negative regulation of transferase activity
neutrophil mediated immunity
gliogenesis
negative regulation of protein kinase activity
regulation of neuron projection development
spindle organization
*granulocyte activation
neutrophil activation involved in immune response
cellular response to ionizing radiation
*neutrophil degranulation
*neutrophil activation
regulation of cell size
regulation of cellular component size
GO term
qscore
Count
20
30
40
50
60
Adjusted p−value
0.03
0.02
0.01
j
Bivalent regions: 4704; H3K4me3−only regions: 11026
H3K27me3−
H3K27me3+
MCIP−seq signal (mean counts)
Location
−1kb
−0.5kb
0kb
0.5kb
1kb
CTCF peaks in CIMP cases
CIMP
AML
T−ALL
CD34
B−cells
T−cells
Granulocytes
k
Bivalent regions: 4704; H3K4me3−only regions: 11026
H3K27me3−
H3K27me3+
MCIP−seq signal
CIMP
AML
T−ALL
CD34
Monocytes
Granulocytes
T−cells
B−cells
l
GSEA C2 collection
Pathway | Gene ranks | NES | pval | padj
MARTENS_TRETINOIN_RESPONSE_UP 1.72 4.7·10^−9 1.9·10^−5
MIKKELSEN_MEF_HCP_WITH_H3K27ME3 1.62 1.3·10^−6 2.6·10^−3
MEISSNER_BRAIN_HCP_WITH_H3K27ME3 1.81 6.0·10^−6 8.1·10^−3
NIKOLSKY_BREAST_CANCER_17Q21_Q...PLICON 1.81 3.0·10^−5 2.7·10^−2
WP_PHYSIOLOGICAL_AND_PATHOLOGI..._HEART 2.02 3.3·10^−5 2.7·10^−2
HELLER_HDAC_TARGETS_DN −1.67 9.1·10^−5 3.0·10^−2
NEDDYLATION −1.60 1.4·10^−3 1.4·10^−1
HSIAO_LIVER_SPECIFIC_GENES −1.62 1.6·10^−3 1.5·10^−1
SWEET_KRAS_ONCOGENIC_SIGNATURE −1.87 1.7·10^−3 1.5·10^−1
TCGA_GLIOBLASTOMA_COPY_NUMBER_UP −1.91 2.2·10^−3 1.5·10^−1
Enriched in CIMP
Enriched in T-ALL
GSEA C5 collection
CELL_SURFACE_RECEPTOR_SIGNALIN...NALING 1.53 1.4·10^−4 6.4·10^−1
CELL_CELL_SIGNALING_BY_WNT 1.58 3.8·10^−4 6.4·10^−1
NEGATIVE_REGULATION_OF_PROTEIN...INDING 1.88 6.9·10^−4 6.4·10^−1
CANONICAL_WNT_SIGNALING_PATHWAY 1.55 7.5·10^−4 6.4·10^−1
INTRINSIC_APOPTOTIC_SIGNALING_...DAMAGE 1.85 1.2·10^−3 6.4·10^−1
REGULATION_OF_PRODUCTION_OF_SM...BY_RNA −2.17 3.6·10^−4 6.4·10^−1
SPECIFIC_GRANULE_MEMBRANE −1.75 9.5·10^−4 6.4·10^−1
DIVALENT_INORGANIC_CATION_HOME...STASIS −1.34 1.6·10^−3 6.4·10^−1
POSITIVE_REGULATION_OF_CELL_DE...OPMENT −1.45 2.3·10^−3 6.4·10^−1
m
BENPORATH_ES_WITH_H3K27ME3 1.62 1.0·10^−50 4.0·10^−47
BENPORATH_SUZ12_TARGETS 1.62 6.3·10^−46 1.3·10^−42
BENPORATH_EED_TARGETS 1.60 2.8·10^−44 3.7·10^−41
BENPORATH_PRC2_TARGETS 1.67 2.8·10^−38 2.8·10^−35
MIKKELSEN_MEF_HCP_WITH_H3K27ME3 1.65 1.4·10^−36 1.1·10^−33
WP_UNFOLDED_PROTEIN_RESPONSE −1.53 1.3·10^−2 3.2·10^−1
METALLOPROTEASE_DUBS −1.26 6.1·10^−2 6.8·10^−1
TRANSPORT_OF_THE_SLBP_DEPENDAN...E_MRNA −1.19 7.9·10^−2 7.3·10^−1
INTERLEUKIN_RECEPTOR_SHC_SIGNALING −1.30 8.5·10^−2 7.5·10^−1
HOFFMANN_PRE_BI_TO_LARGE_PRE_B...YTE_UP −1.13 1.1·10^−1 7.8·10^−1
Enriched in CIMP
Enriched in CD34
DNA_BINDING_TRANSCRIPTION_FACT...TIVITY 1.44 5.6·10^−29 3.9·10^−25
CHROMATIN 1.44 5.0·10^−24 1.7·10^−20
TRANSCRIPTION_REGULATOR_ACTIVITY 1.36 7.2·10^−24 1.7·10^−20
CIS_REGULATORY_REGION_SEQUENCE...INDING 1.42 3.0·10^−23 5.3·10^−20
SEQUENCE_SPECIFIC_DNA_BINDING 1.37 1.8·10^−22 2.6·10^−19
ENDOPLASMIC_RETICULUM_TUBULAR_...ETWORK −2.06 2.4·10^−3 4.6·10^−2
PERICENTRIC_HETEROCHROMATIN −1.53 2.0·10^−2 1.8·10^−1
CYTOPLASMIC_TRANSLATIONAL_INIT...IATION −1.38 4.7·10^−2 2.7·10^−1
LYSINE_ACETYLATED_HISTONE_BINDING −1.46 1.1·10^−1 4.4·10^−1
MEMBRANE_LIPID_CATABOLIC_PROCESS −1.36 1.2·10^−1 4.5·10^−1
n
TLE4
KITLG
FGF9
2238
2668
DD82
7076
6376
7120
AML0101
AML0102
UKRT1
UKRT2
UKRT3
UKRT4
~82177.2 Kb chr9 ~82198.7 Kb
~88963.3 Kb chr12 ~88984.8 Kb
~22232.9 Kb chr13 ~22255.7 Kb
CIMP
AML
T−ALL

Supplementary Figure 5. CIMP leukemias exhibit hypermethylation at regulatory regions. Unless otherwise specified, methylation was measured by MCIP-seq with the following groups and sample sizes: CIMP (n=13), AML (n=50), T-ALL (n=14) and CD34+ cells (n=3). Panels **b**, **j** and **k** also include data from healthy granulocytes, monocytes, T-cells and B-cells (n=2 each). **a** Box plots displaying methylation levels of leukemias and CD34+ cells at genomic regions of the Blueprint regulatory build [5]. The lower and upper edges represent the first and third quartiles, respectively; the horizontal line indicates the median. The whiskers extend to the most extreme values within the range comprised between the median and 1.5 times the interquartile range. **b** Same as **a**, but at genomic features defined relative to gene features. **c** Average methylation levels in a 4-kb window around the center of putative promoters. **d** Tornado plot depicting methylation levels at putative promoters in CIMP leukemias and healthy cells. The HSPC tracks in purple were downloaded from ENCODE [6] and show chromatin accessibility (DNase-seq) as well as histone marks for enhancers (H3K4me1), promoters (H3K4me3), activation (H3K27ac) and repression (H3K27me3). GC density was downloaded from the UCSC browser [7]. **e** Violin plot showing methylation levels of AML (n=272), T-ALL (n=119), ETP-ALL (n=24) and CIMP leukemias (n=5) at different genomic features measured by MethylationEPIC array. **f** Number of differentially methylated regions (DMRs) detected by supervised comparisons of MethylationEPIC array data. CpG sites with absolute mean beta value difference > 0.2 and FDR-adjusted p-value < 0.05 were considered differentially methylated. **g-i** Dot plots of GO term enrichment on genes overlapping DMRs obtained from MethylationEPIC array data. **j** Average methylation levels in 1-kb windows around the center of H3K4me1 peaks, either overlapping H3K27me3 peaks (bivalent regions, right) or not (left). **k** Box plots depicting the same data as in j, but from the central 100 bp of each peak. See a for a definition of the box plot components. **l** Summary of the top 5 most significant results of pre-ranked GSEA conducted on genes in the vicinity of DMRs between CIMP and T-ALL. The C2 (left) and C5 (right) MSigDB collections were used in the analysis. **m** Same as **l**, but showing enrichment in CIMP relative to CD34+ cells. **n** Genomic tracks of MCIP-seq data for selected leukemia samples at gene promoters with significant changes in methylation.

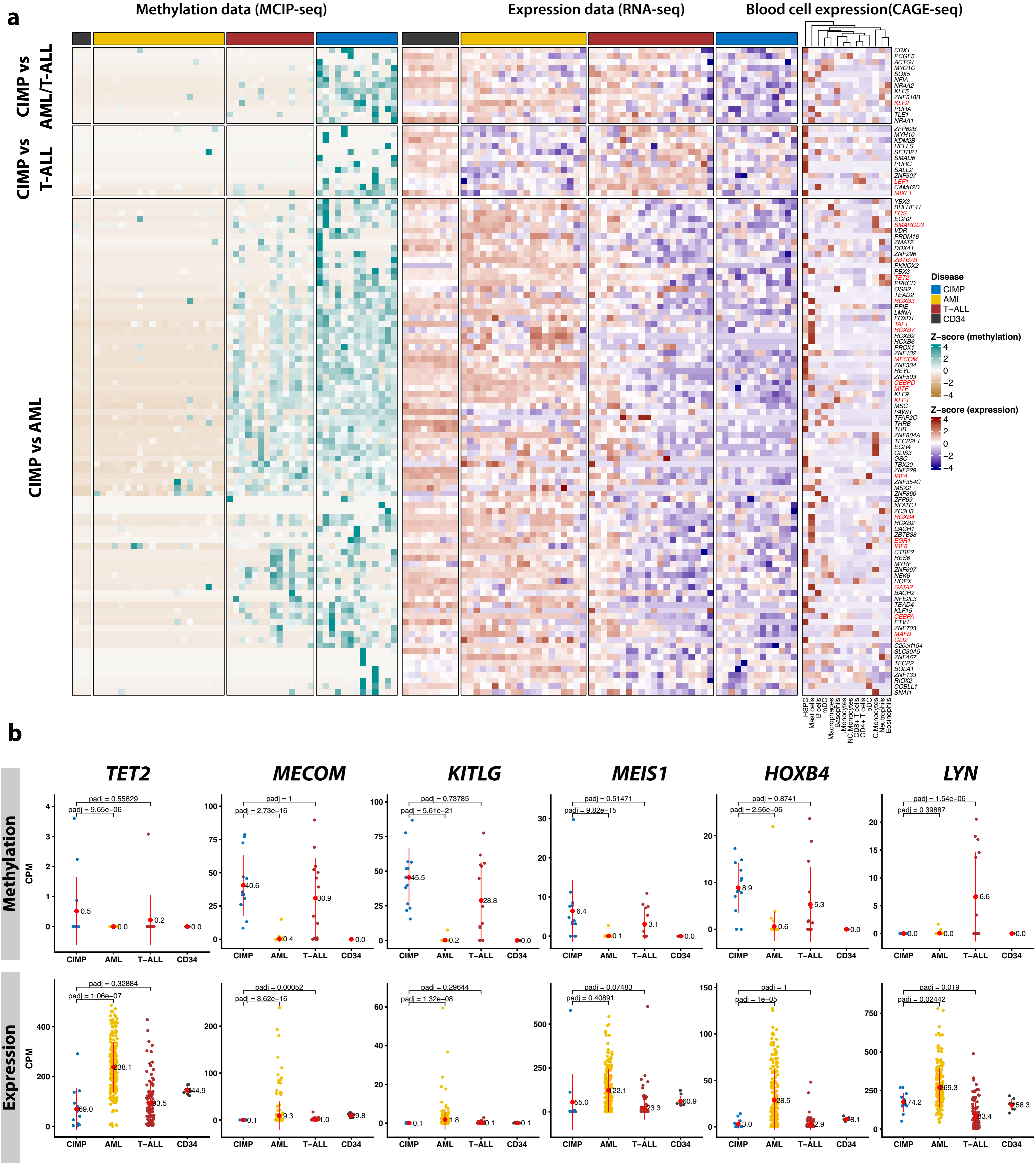

a
Methylation data (MCIP-seq)
Expression data (RNA-seq)
Blood cell expression(CAGE-seq)
CIMP vs AML/T-ALL
CIMP vs T-ALL
CIMP vs AML
Disease
CIMP
AML
T-ALL
CD34
Z-score (methylation)
Z-score (expression)
HSPC
Mast cells
B cells
mDC
Macrophages
Basophils
I.Monocytes
NC.Monocytes
CD8+ T cells
CD4+ T cells
pDC
C.Monocytes
Neutrophils
Eosinophils
b
TET2
MECOM
KITLG
MEIS1
HOXB4
LYN
Methylation
Expression
CPM
CIMP
AML
T-ALL
CD34
padj = 0.55829
padj = 9.65e−06
padj = 1
padj = 2.73e−16
padj = 0.73785
padj = 5.61e−21
padj = 0.51471
padj = 9.82e−15
padj = 0.8741
padj = 2.56e−06
padj = 1.54e−06
padj = 0.39887
padj = 0.32884
padj = 1.06e−07
padj = 0.00052
padj = 8.62e−16
padj = 0.29644
padj = 1.32e−08
padj = 0.07483
padj = 0.40891
padj = 1
padj = 1e−05
padj = 0.019
padj = 0.02442

c

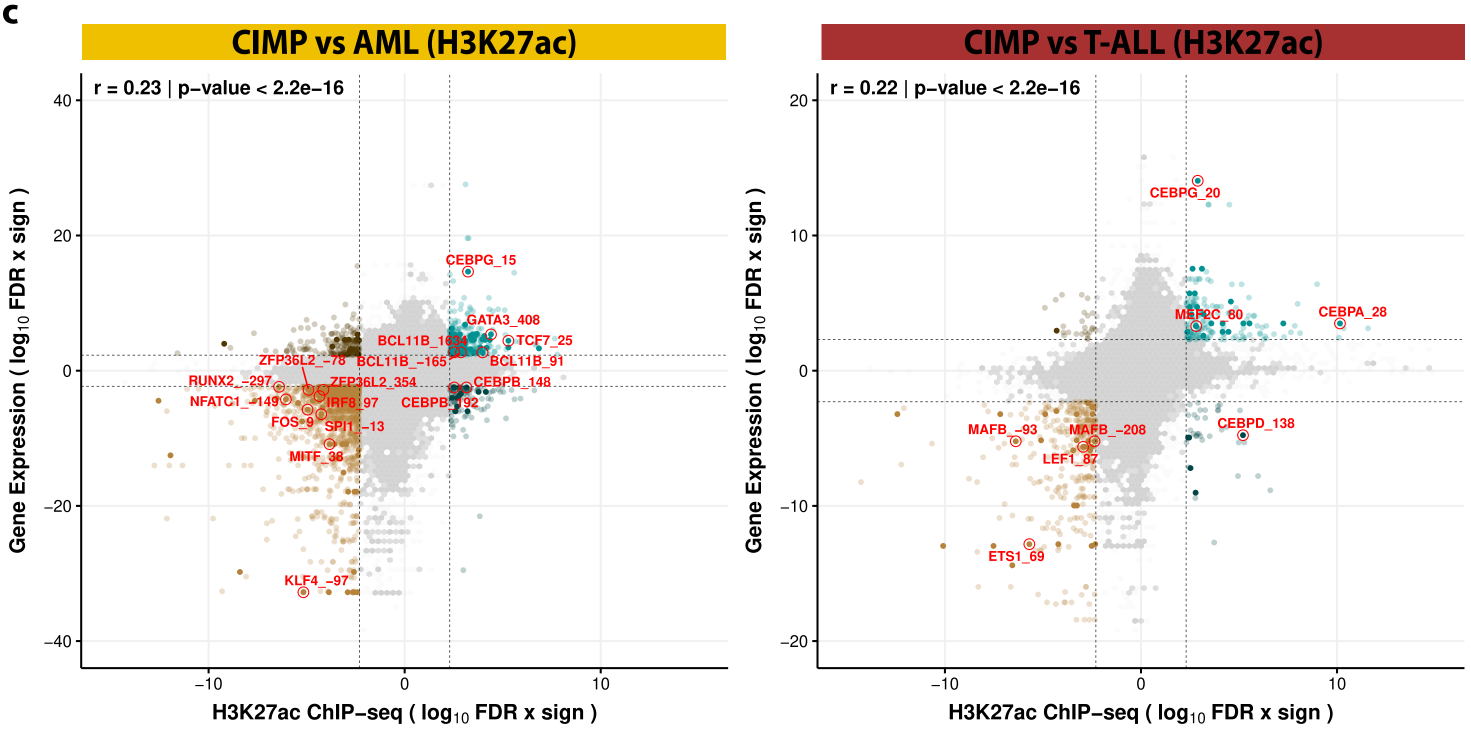

Supplementary Figure 6. Integration of methylation and gene expression data reveals widespread silencing of transcription factors in CIMP. a Heatmap displaying normalized methylation levels (MCIP-seq) at promoters of genes encoding for hematopoietic TFs exhibiting both hypermethylation and loss of expression in CIMP with respect to AML (CIMP vs AML), T-ALL (CIMP vs T-ALL) or both (CIMP vs AML & T-ALL). The heatmap in the middle shows normalized expression levels (RNA-seq) of the same genes in leukemia cells and healthy HSPCs. The rightmost heatmap presents normalized gene expression (CAGE-seq) in different healthy cells. **b** Jitter plots showing methylation (top) and expression (bottom) of a few selected genes in CIMP, other leukemias and CD34+ HSPCs. The horizontal black lines represent pairwise comparisons between CIMP and other leukemias. Statistical significance was determined by a two-sided Wald test in the DESeq2 package and corrected for multiple testing with the Benjamini–Hochberg procedure. Sample sizes for MCIP-seq and RNA-seq were, respectively: 13/13 (CIMP), 50/211 (AML), 14/100 (T-ALL). **c** Starbust plot depicting changes in gene expression (Y-axis) and H3K27ac ChIP-seq at enhancers (X-axis) between CIMP and AML (left) and T-ALL (right). The statistical significance of the comparisons bet ween these groups was determined by the Wald test (two-sided) in the DESeq2 package and corrected for multiple testing with the Benjamini–Hochberg procedure. The values are the log10 of the false discovery rate (FDR) with the sign of the fold change. Genes with FDR < 0.05 and log2 fold change > 2 for both data types are colored (turquoise if hypermethylated, brown if hypomethylated). Those with non-significant changes are binned into grey hexagons whose opacity is proportional to the number of genes therein. Genes encoding for transcription factors (TFs) are shown in solid color, among which those involved in hematopoiesis are highlighted in red (GO term: 0030097) and labeled. The rest of the genes are semitransparent. The Pearson correlation coefficient (r) and its related p-value (two-sided) for the relationship between methylation and expression are shown at the top left. Note that a single gene may be targeted by multiple enhancers, each of which is labeled based on the distance with respect to the TSS. Sample sizes for H3K27ac ChIP-seq and RNA-seq data were, respectively: 9/13 (CIMP), 51/211 (AML) and 19/100 (T-ALL).

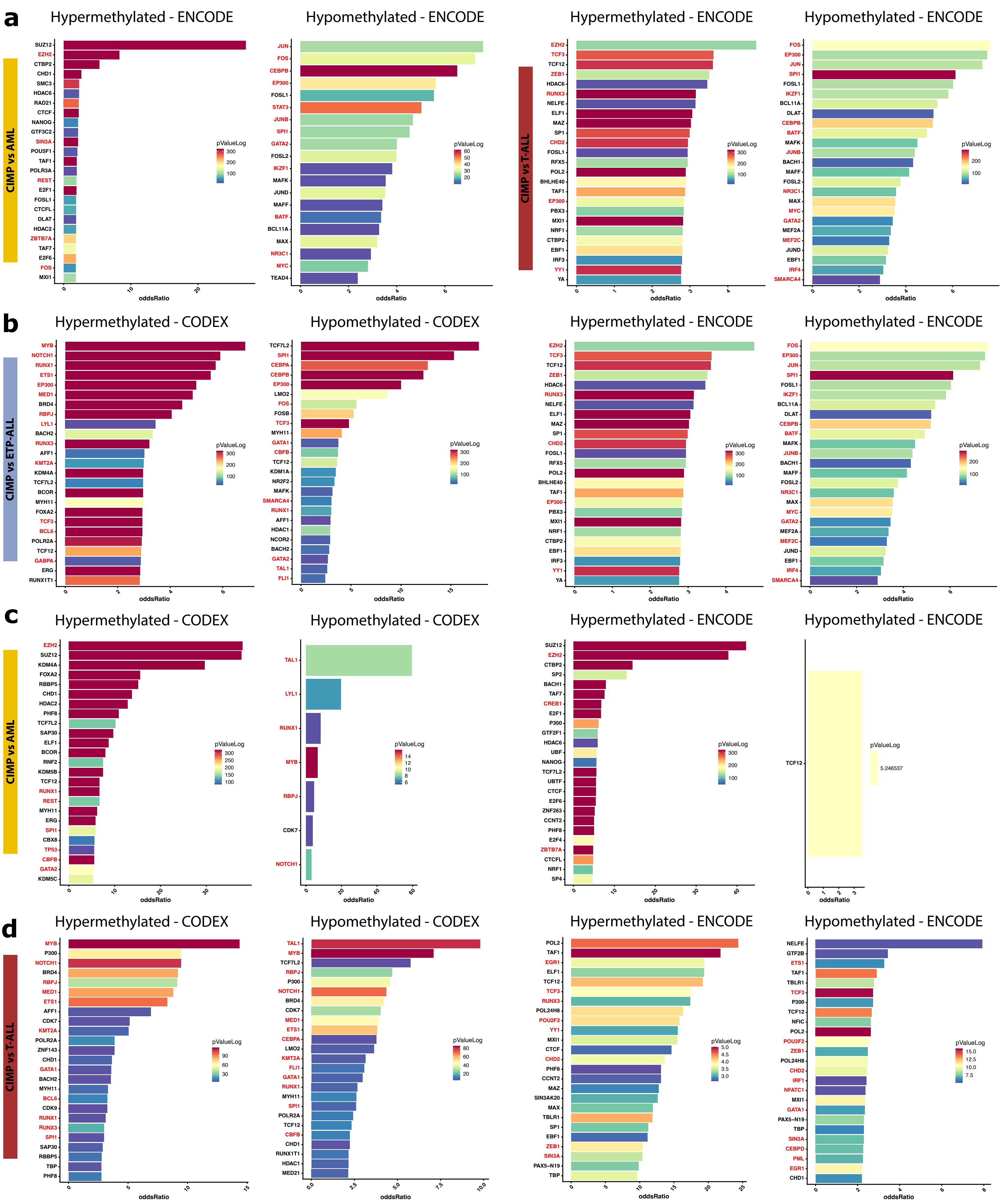

Supplementary Figure 7. Locus overlap analyses of methylation data indicate alterations in binding sites for key hematopoietic factors. a Bar plot depicting enrichment for experimentally confirmed TF binding sites from the ENCODE database at differentially methylated regions between CIMP and AML (left) or between CIMP and T-ALL (right), as derived from EPIC array data. Enrichment was calculated with a two-tailed Fisher's exact test using the LOLA R package. The length of the bars corresponds to the odds ratio and their color to the -log10(p-value); only a maximum of 25 results with a -log(p-value) above 50 are shown. TFs involved in hematopoiesis are highlighted in red. TFs involved in hematopoiesis (GO term: 0030097) are highlighted in red. **b** Same as **a**, but comparing CIMP and ETP-ALL and using both CODEX (top) and ENCODE (bottom) databases to calculate enrichment. **c** Same as **a**, but using MCIP-seq data to identify differentially methylated regions and the CODEX database to determine enrichment. **d** Same as **c**, but the enrichment was calculated with the ENCODE database.

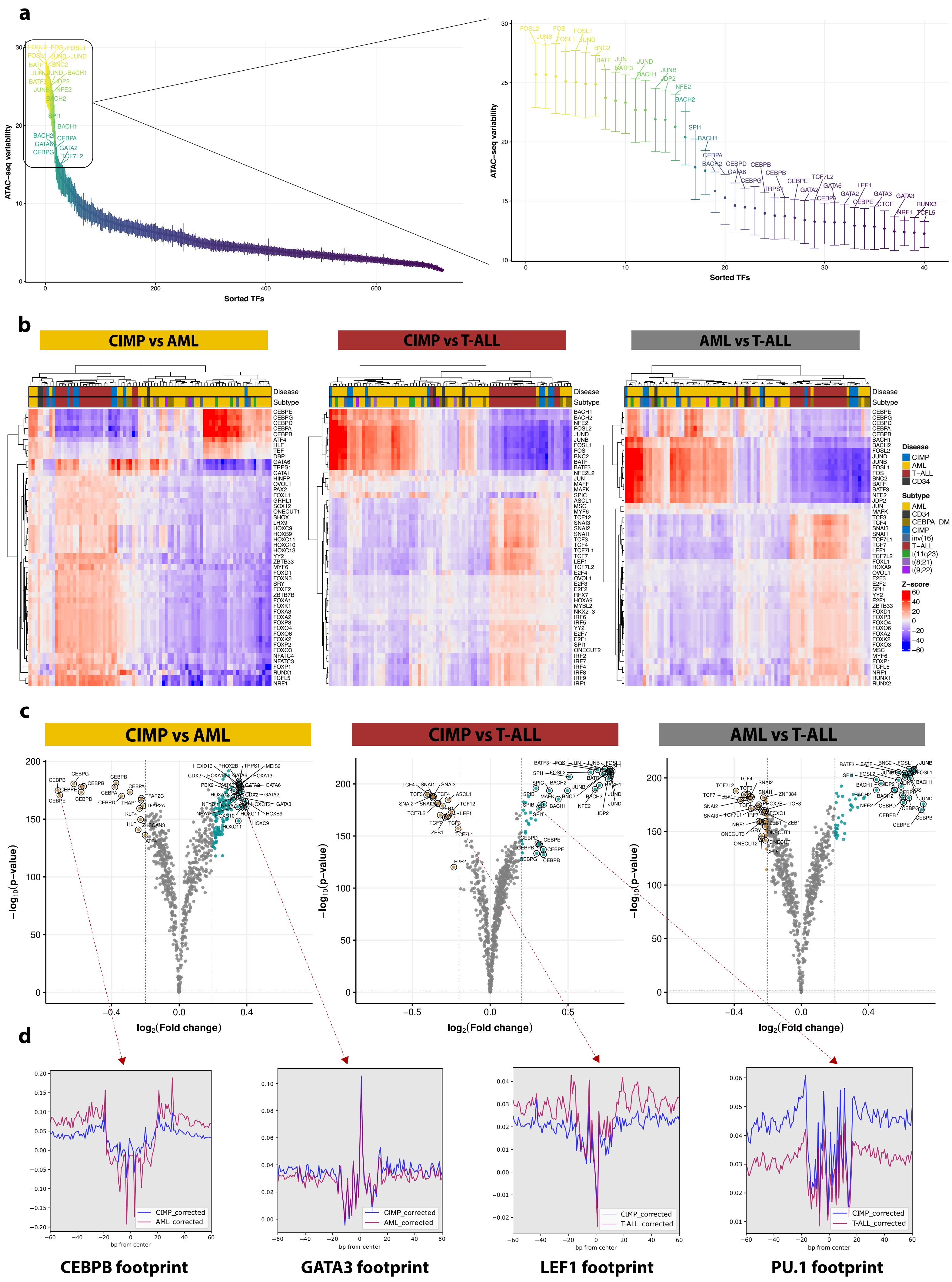
a
ATAC–seq variability
Sorted TFs
b
CIMP vs AML
CIMP vs T-ALL
AML vs T-ALL
Disease
Subtype
CIMP
AML
T–ALL
CD34
CEBPA_DM
inv(16)
t(11q23)
t(8;21)
t(9;22)
Z–score
c
CIMP vs AML
CIMP vs T-ALL
AML vs T-ALL
−log10(p−value)
log2(Fold change)
d
CIMP_corrected
AML_corrected
T-ALL_corrected
bp from center
CEBPB footprint
GATA3 footprint
LEF1 footprint
PU.1 footprint

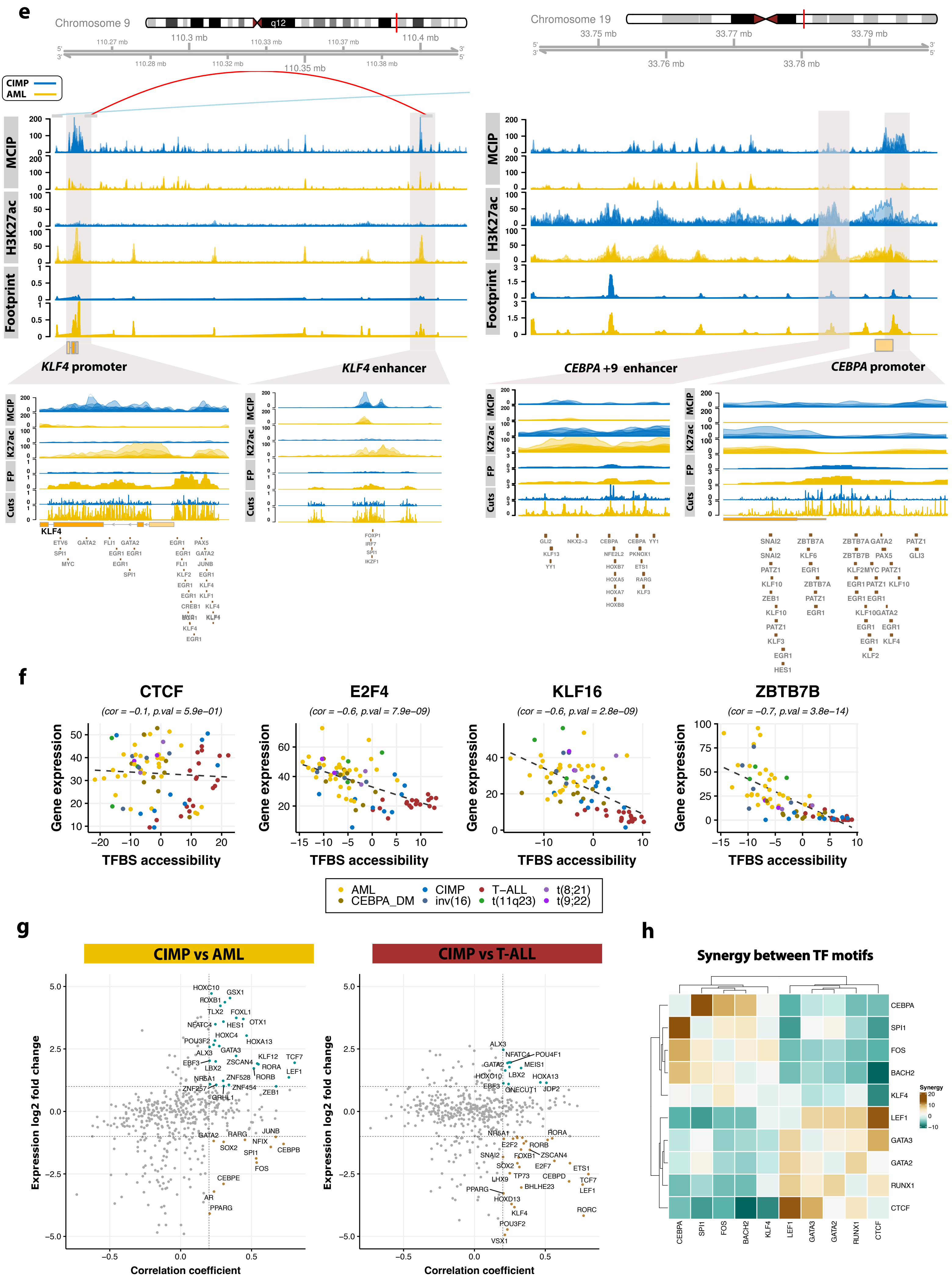
e
Chromosome 9
q12
110.27 mb
110.3 mb
110.33 mb
110.37 mb
110.4 mb
110.28 mb
110.32 mb
110.35 mb
110.38 mb
CIMP
AML
MCIP
H3K27ac
Footprint
Chromosome 19
33.75 mb
33.77 mb
33.79 mb
33.76 mb
33.78 mb
KLF4 promoter
KLF4 enhancer
CEBPA +9 enhancer
CEBPA promoter
MCIP
K27ac
FP
Cuts
KLF4
f
CTCF
(cor = −0.1, p.val = 5.9e−01)
E2F4
(cor = −0.6, p.val = 7.9e−09)
KLF16
(cor = −0.6, p.val = 2.8e−09)
ZBTB7B
(cor = −0.7, p.val = 3.8e−14)
Gene expression
TFBS accessibility
AML
CEBPA_DM
CIMP
inv(16)
T−ALL
t(11q23)
t(8;21)
t(9;22)
g
CIMP vs AML
CIMP vs T-ALL
Expression log2 fold change
Correlation coefficient
h
Synergy between TF motifs
Synergy
CEBPA
SPI1
FOS
BACH2
KLF4
LEF1
GATA3
GATA2
RUNX1
CTCF

Supplementary Figure 8. Estimation of motif activity based on open chromatin data. a Transcription factor motifs ranked by variability in chromatin accessibility, as measured by chromVar using ATAC-seq data. The top 40 are shown as a zoom-in underneath. **b** Heatmaps displaying the individual-specific motif activities, as Z-score, of the top 50 TFs with highest differential activity between CIMP and AML (left), CIMP and T-ALL (middle), AML and T-ALL (right). The activity of motifs corresponding to the same TF was averaged. **c** Volcano plot showing the results of the TF footprinting analysis conducted with TOBIAS, aggregating data from all CIMP patients (n=9), AMLs (n=20) and T-ALL (n=20). The top 30 most significant results are highlighted and labelled. **d** Aggregated ATAC-seq signal across all motif occurrences of 4 TFs exhibiting significantly different footprints between CIMP and either AML (CEBPB, GATA3) or T-ALL (LEF1, PU.1). **e** Genomic tracks displaying footprint scores calculated by TOBIAS in the regulatory regions of KLF4 and CEBPA, as well as motifs identified in those genomic locations. H3K27ac data are shown to indicate the presence of putative enhancers or promoters, whereas MCIP reveals hypermethylation at inaccessible regions. **f** Correlation between motif activity as estimated by chromVAR and TPM-normalized gene expression in every individual where both RNA-seq and ATAC-seq data were available (n=79). The scatter plots show relevant TFs not included in **Figure 5d**; the full dataset is available in Supplementary Data 34. **g** Scatter plot identifying differentially expressed regulators between CIMP and AML (left) or T-ALL (right). The X-axis shows the correlation coefficient between accessibility and gene expression across the entire cohort (positive regulators have high correlation), whereas the Y-axis shows differential gene expression between two leukemia groups. The color points are significant positive regulators, defined by rho ≥ 0.2 and log2(fold change) ≥ 1. **h** Heatmap displaying synergy between a subset of variable TFs, defined as the deviation of chromatin accessibility in peaks with both motifs relative to peaks with only one motif. A high synergy score can indicate cooperativity or competition between TFs.

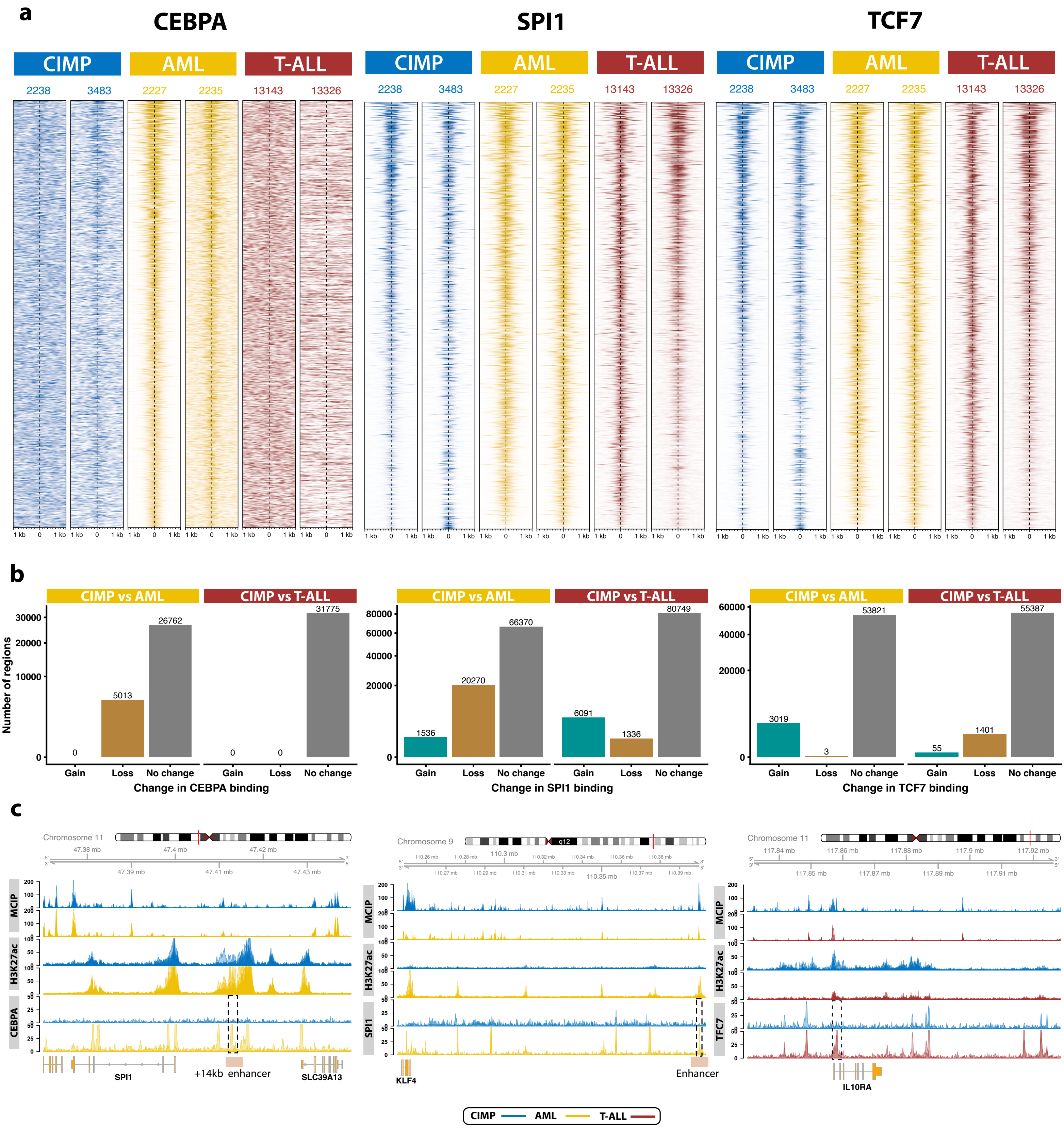

Supplementary Figure 9. CIMP leukemias exhibit dysregulated patterns of transcription factor binding. a Tornado plot depicting the signal of three TFs (CEBPA, SPI1 and TCF7 from left to right) in the 5000 most variable peaks in CIMP, AML and T-ALL (n=2 per group). The peaks are sorted based on total signal across all samples, from higher to lower. **b** Bar plots showing the number of differential peaks between CIMP and either AML or T-ALL for each TF (CEBPA, SPI1 and TCF7, left to right). Only peaks with p-value < 0.01 and log2 (fold change) > 1 are shown. **c** Genomic tracks of selected loci with differential TF binding. Left: binding of CEBPA is lost at a +14 kb enhancer of SPI1, middle: CIMP cases exhibit loss of SPI1 binding at a KLF4 enhancer, compared to AML; right: TCF7 binds the IL10RA in T-ALL, but not in CIMP leukemias.

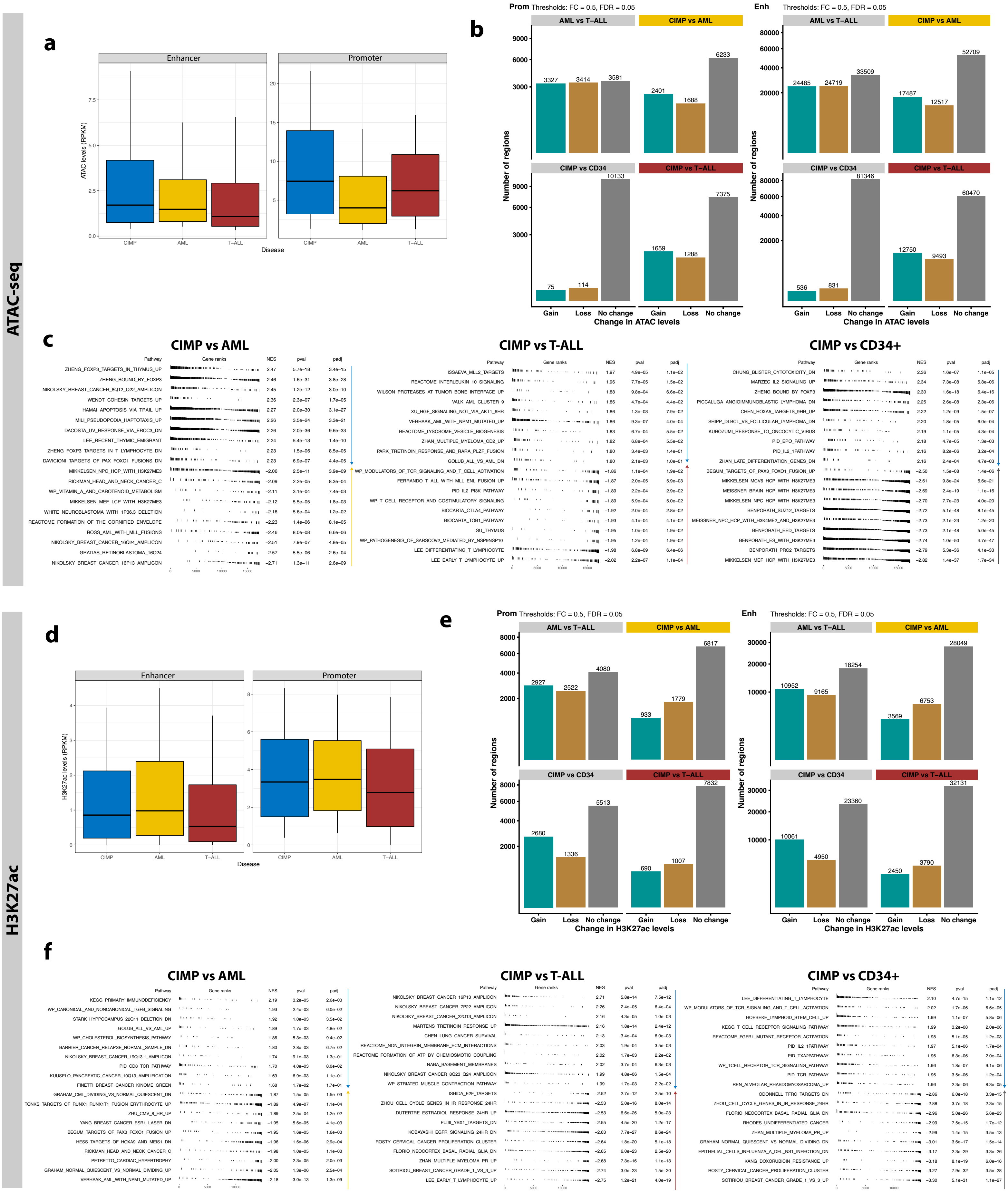
ATAC-seq
a
Enhancer
Promoter
ATAC levels (RPKM)
CIMP
AML
T–ALL
Disease
b
Prom Thresholds: FC = 0.5, FDR = 0.05
Enh Thresholds: FC = 0.5, FDR = 0.05
AML vs T–ALL
CIMP vs AML
CIMP vs CD34
CIMP vs T–ALL
Number of regions
3327
3414
3581
2401
1688
6233
75
114
10133
1659
1288
7375
24485
24719
33509
17487
12517
52709
536
831
81346
12750
9493
60470
Gain
Loss
No change
Change in ATAC levels
c
CIMP vs AML
CIMP vs T-ALL
CIMP vs CD34+
Pathway
Gene ranks
NES
pval
padj
H3K27ac
d
Enhancer
Promoter
H3K27ac levels (RPKM)
CIMP
AML
T–ALL
Disease
e
Prom Thresholds: FC = 0.5, FDR = 0.05
Enh Thresholds: FC = 0.5, FDR = 0.05
AML vs T–ALL
CIMP vs AML
CIMP vs CD34
CIMP vs T–ALL
Number of regions
2927
2522
4080
933
1779
6817
2680
1336
5513
690
1007
7832
10952
9165
18254
3569
6753
28049
10061
4950
23360
2450
3790
32131
Gain
Loss
No change
Change in H3K27ac levels
f
CIMP vs AML
CIMP vs T-ALL
CIMP vs CD34+
Pathway
Gene ranks
NES
pval
padj

H3K27me3

g

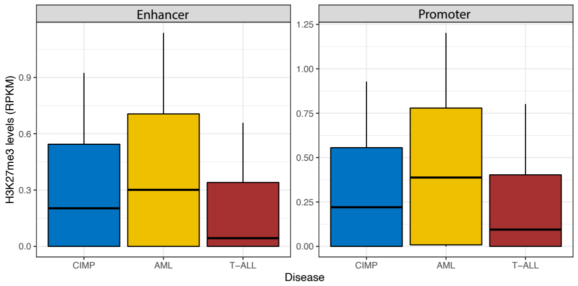

h

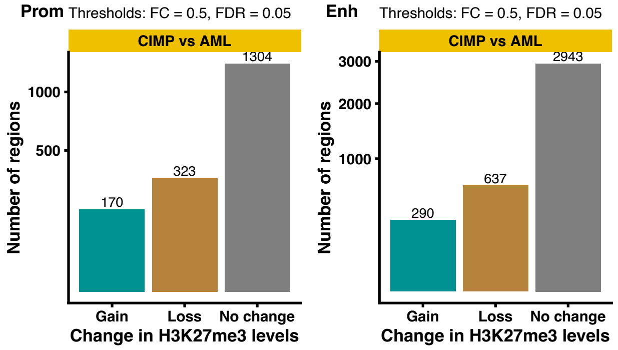

i

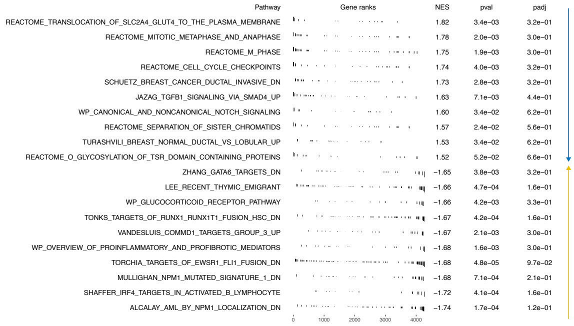

Supplementary Figure 10. Additional analyses of epigenomics data. a Box plots displaying open chromatin levels (ATAC-seq) computed by DiffBind at enhancers and promoters for CIMP (n=9), AML (n=51) and T –ALL (n=19). The lower and upper edges of the boxplots represent the first and third quartiles, respectively; the horizontal line inside the box indicates the median. The whiskers extend to the most extreme values within the range comprised between the median and 1.5 times the interquartile range. **b** Bar plot of differentially accessible regions in various supervised comparisons. A threshold of FDR < 0.05 and |log2 FC| > 0.5 was used to determine significance. **c** Bar plot showing the top results (10 highest and 10 lowest) from gene set enrichment analysis (GSEA) conducted on genes close to open chromatin peaks, using the C2 collection. The ranking of genes was based on differential chromatin accessibility for each comparison, i.e. CIMP vs AML (left), CIMP vs T-ALL (middle) and CIMP vs CD34+ cells (right). **d-f** Same as **a-c**, but using H3K27ac ChIP-seq data instead in CIMP (n=9), AML (n=51) and T-ALL (n=19). **g-i** Same as **a-c**, but using H3K27me3 data instead in CIMP (n=5) and AML (n=22).

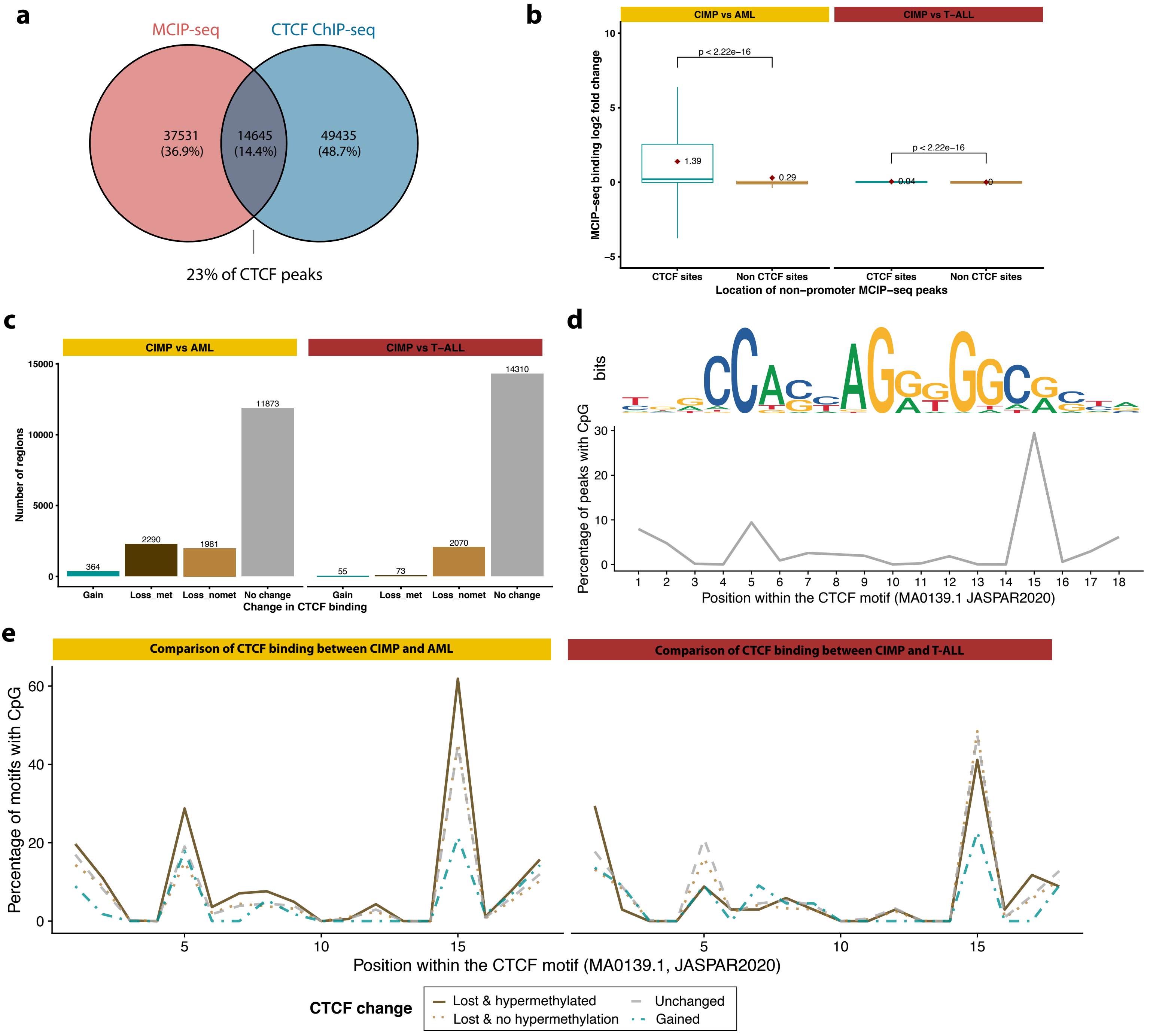

Supplementary Figure 11. Effects of methylation on CTCF binding in CIMP and other leukemias. a Venn diagram with overlap between the consensus lists of MCIP-seq peaks and CTCF ChIP-seq peaks. **b** Box plot displaying changes in methylation between CIMP and AML (left) or T-ALL (right) at MCIP-seq peaks that either overlap with CTCF binding sites or do not. The lower and upper edges of the boxplots represent the first and third quartiles, respectively; the horizontal line inside the box indicates the median. The whiskers extend to the most extreme values within the range comprised between the median and 1.5 times the interquartile range. **c** Number of CTCF binding sites that are gained (Gain), lost with hypermethylation (Lost_met), lost without change in methylation (Loss_nomet) or unchanged in comparisons between CIMP and AML. Only regions with data from both -CTCF ChIP-seq and MCIP-seq are considered. **d** Average frequency of CpG dinucleotides in all detected CTCF binding sites at every position of the CTCF motif (MA0139.1, JASPAR database7). **e** Same as **d**, but average CpG frequencies are calculated for the fractions of differential CTCF peaks described in **c**. Sample sizes for CTCF ChIP-seq in CIMP, AML and T-ALL were 9, 10 and 19 respectively**.**

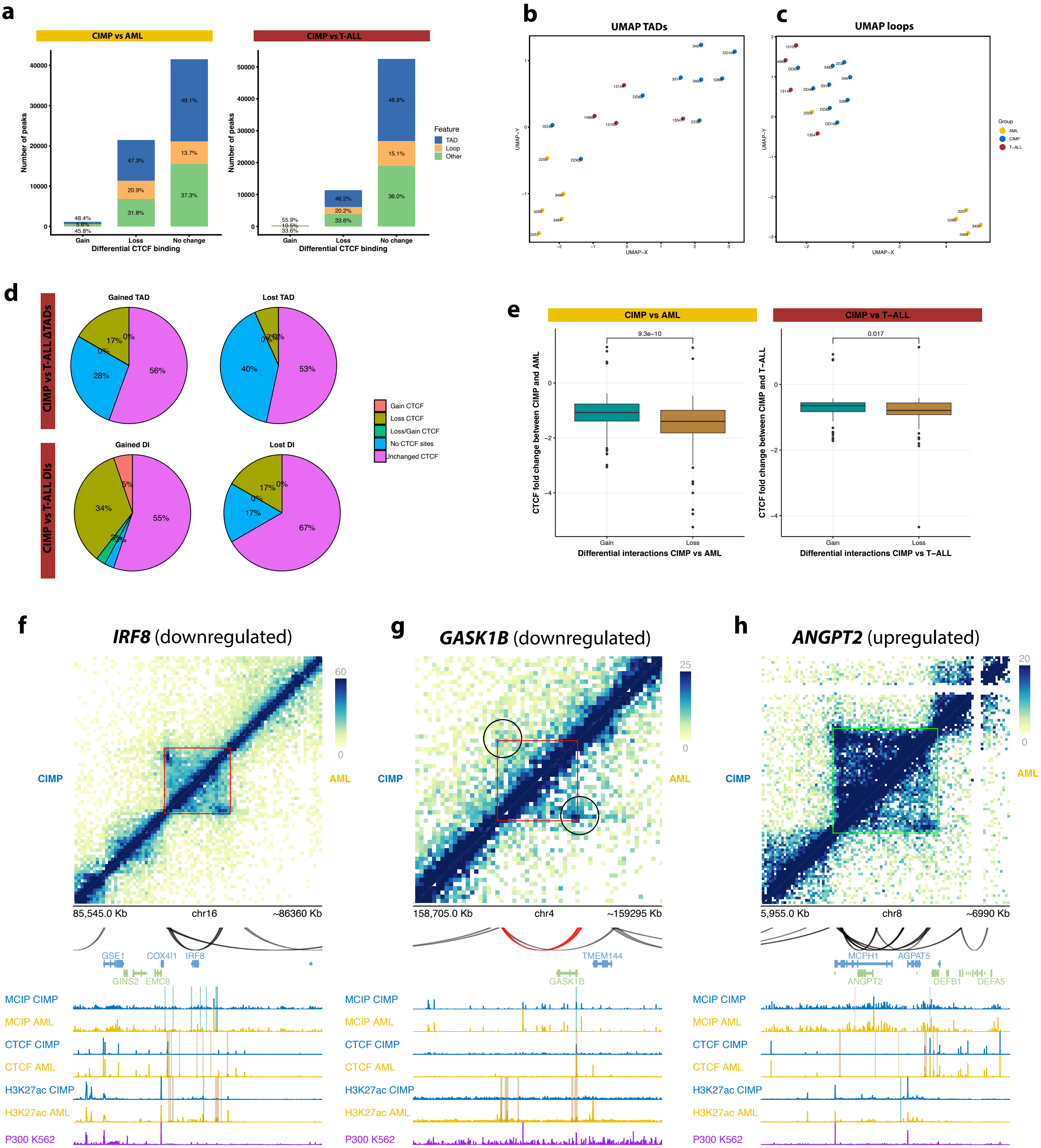
a
CIMP vs AML
CIMP vs T-ALL
Number of peaks
Differential CTCF binding
Gain
Loss
No change
Feature
TAD
Loop
Other
b
UMAP TADs
c
UMAP loops
UMAP-X
UMAP-Y
Group
AML
CIMP
T-ALL
d
CIMP vs T-ALL ΔTADs
CIMP vs T-ALL ΔDIs
Gained TAD
Lost TAD
Gained DI
Lost DI
Gain CTCF
Loss CTCF
Loss/Gain CTCF
No CTCF sites
Unchanged CTCF
e
CIMP vs AML
CIMP vs T-ALL
9.3e−10
0.017
CTCF fold change between CIMP and AML
CTCF fold change between CIMP and T-ALL
Differential interactions CIMP vs AML
Differential interactions CIMP vs T-ALL
f
IRF8 (downregulated)
g
GASK1B (downregulated)
h
ANGPT2 (upregulated)
CIMP
AML
85,545.0 Kb
chr16
~86360 Kb
158,705.0 Kb
chr4
~159295 Kb
5,955.0 Kb
chr8
~6990 Kb
GSE1
COX4I1
IRF8
GINS2
EMC8
TMEM144
GASK1B
MCPH1
AGPAT5
ANGPT2
DEFB1
DEFA5
MCIP CIMP
MCIP AML
CTCF CIMP
CTCF AML
H3K27ac CIMP
H3K27ac AML
P300 K562

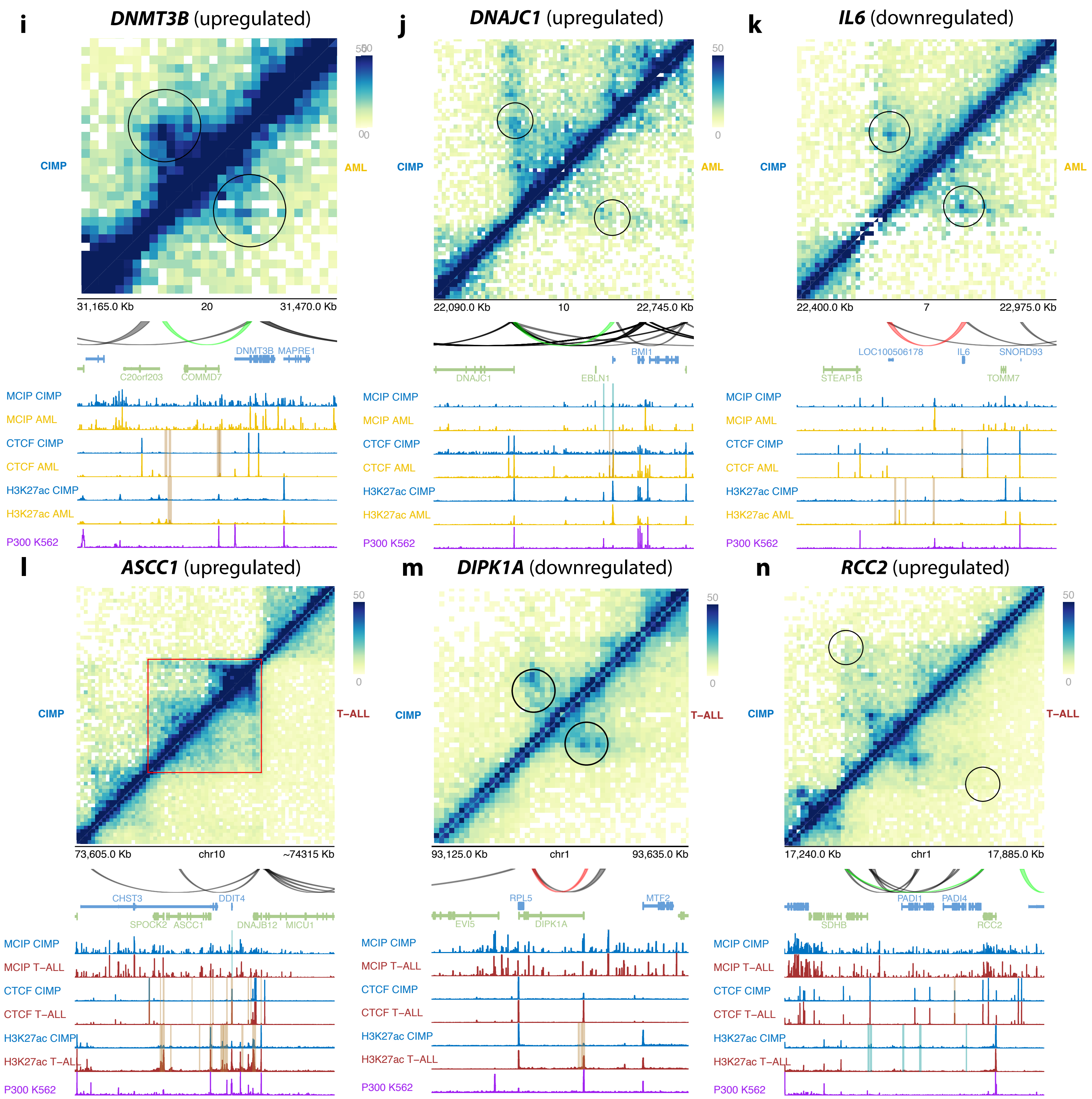

Supplementary Figure 12. Differences in 3D genome organization between leukemia groups. Differential analyses of Hi-C data in panels **d-g** were performed between CIMP (n=8) and either AML (n=5) or T-ALL (n=4). **a** Bar plots depicting the percentage of differential CTCF peaks that overlap with TAD boundaries or loop anchors. **b** UMAP plot of TAD inclusion ratios (IR) calculated by HOMER in Hi-C data. **c** UMAP plot of loop density scores calculated by HOMER in Hi-C data. **d** Distribution of gains or losses in CTCF binding in variable TADs (top) or differential interactions (bottom) when comparing CIMP vs T-ALL. **e** Box plot showing the change in CTCF binding (expressed as log2) at gained or lost differential interactions between CIMP and AML (left) or T-ALL (right). The lower and upper edges of the boxplots represent the first and third quartiles, respectively; the horizontal line indicates the median. The whiskers extend to the most extreme values within the range between the median and 1.5 times the interquartile range. For the CTCF ChIP-seq analysis, we used 9 CIMP, 10 AML and 19 T-ALL. **f** Merged Hi-C contact map of the *IRF8* locus, comparing interactions between the CIMP (uppermost triangle, n=5) and AML groups (bottom triangle, n=5). ΔTADs are highlighted as squares, colored in green if insulation is gained or in red if insulation is lost; DIs are indicated with black circles. Underneath, all loops detected in this region are shown in black, if they are invariable across conditions, and in green or red if they are gained or lost in CIMP relative to AML, respectively. The tracks below display MCIP-seq, CTCF ChIP-seq and H3K27ac ChIP-seq from CIMP and AML (n=4). Peaks gained in CIMP are highlighted in turquoise, whereas lost peaks are highlighted in light brown. The last track shows p300 binding measured by ChIP-seq in the K562 cell line. **g-k** Same as **f**, but the *GASK1B, ANGPT2, DNMT3B, IL6* and *DNAJC1* loci are shown, respectively. **l-n** Same as **f**, but for comparisons between CIMP (n=4) and T-ALL (n=4). The *ASCC1*, *DIPK1A* and *RCC2* loci are shown, respectively.

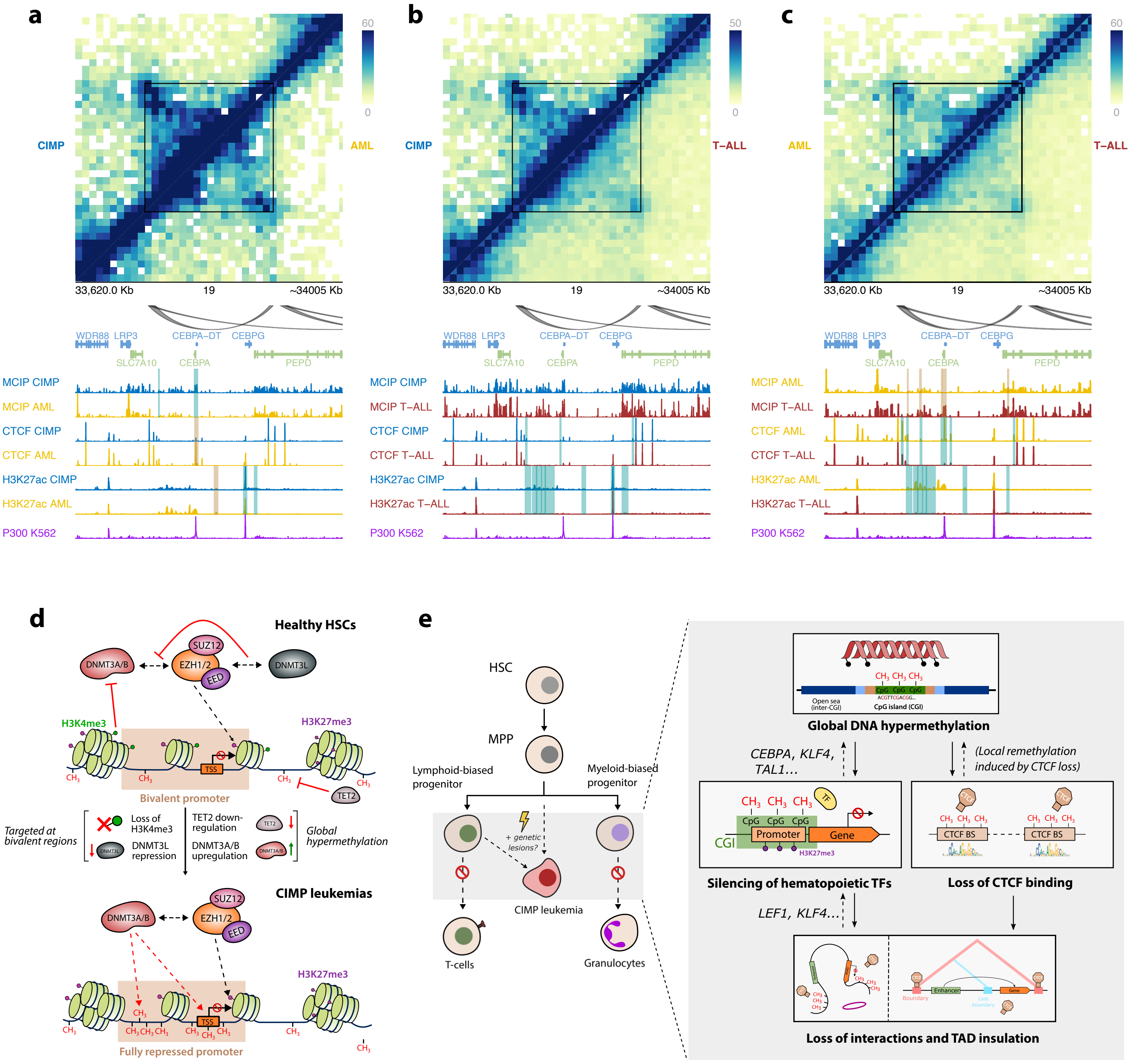

Supplementary Figure 13. Additional figures for discussion. a-c Merged Hi-C contact maps of the *CEBPA* locus, comparing interactions between CIMP and AML (**a**), CIMP and T-ALL (**b**), AML and T-ALL (**c**). The *CEBPA* TAD is indicated as a black square. Underneath, all loops detected in this region are shown in black, if they are invariable across conditions, and in green or red if they are gained or lost, respectively. The tracks below display aggregated MCIP-seq, CTCF ChIP-seq and H3K27ac ChIP-seq data (n=4 each). Peaks gained are highlighted in turquoise, whereas lost peaks are highlighted in light brown. The last track shows p300 binding measured by ChIP-seq in the K562 cell line. **d** Proposed mechanism of preferential hypermethylation at H3K27ac-marked regions. In CIMP leukemias, lack of DNMT3L and loss of H3K4me3 at bivalent regions enables the binding of DNMT3 proteins, recruited by EZH2. Moreover, the lack of TET2 prevents active demethylation. **e** Diagram summarizing the epigenetic mechanisms described in this study leading to differentiation block in CIMP leukemias. Aberrant DNA hypermethylation represses transcription of key hematopoietic TFs (notably *CEBPA)* and disrupts the binding of TFs genome-wide. In particular, the loss of CTCF binding at TAD boundaries and loop anchors results in local 3D genome reorganization, which is also influenced by silencing of TFs like *LEF1* or *KLF4*, known to be involved in chromatin remodelling. Altogether, these processes rewire regulatory networks driving hematopoietic differentiation in early progenitors, leaving them unable to fully commit to either the lymphoid or the myeloid lineage.

SUPPLEMENTARY RESULTS

The mutational landscape of CIMPs is similar to that of ETP-ALL

Although CIMP cases were originally diagnosed as AML, the mixed lymphoid and myeloid phenotype of these leukemias calls for a revised classification. To evaluate their relationship with other leukemias at the genetic level, we compiled data from published studies on AML [9,10], T-ALL [11–14], ETP-ALL [2,13–16] and T/M MPAL [17,18] (Supplementary Data 6-7). Genes like *NOTCH1*, *PHF6*, *JAK3* or *DNM2* were more often mutated in CIMP than in AML (p-value < 0.05, two-tailed Fisher's exact test), but not than in T-ALL, whereas *DNMT3A*, *IDH2* and *RUNX1* exhibited mutation frequencies more similar to AML than to T-ALL. Mutation frequencies were generally comparable to T/M MPAL and especially ETP-ALL, although this assessment might be limited by the small sample size of the CIMP cohort. A significant difference was found for *MED12*, mutated in 36% of the CIMP cases, only 5% of all ETP-ALL and 1% of T/M MPAL. Interestingly, only two CIMP patients harbored lesions in *CHEK2*, a gene involved in DNA damage response. Although rare in *de novo* leukemia, germline *CHEK2* mutations have been reported to increase predisposition to various hematological malignancies, often following treatment for solid cancer [19–22]. Data on gene fusion were less conclusive, as no recurrent fusions were identified in CIMP cases and studies in other cohorts yielded contradictory results (Supplementary Data 8).

The frequency of CNAs in CIMPs (average, 13/genome) was higher than in AML (average, 6.3/genome), but lower than in T-ALL (average, 24.1/genome). However, aneuploidies were found much more frequently in the CIMP group (7/14) than in both AML (5/44) and T-ALL (5/14). This fraction was also much larger than the 20% of AML with aneuploidies reported in the literature [23], and frequent CNAs observed in AML, such as gain of chr8 or loss of chr7, were not particularly overrepresented in this cohort [24,25]. Altogether, this suggests unexpected genetic instability in these leukemias, which may be linked to genetic or epigenetic alterations in genes involved in DNA repair.

Even though no single gene was mutated in all CIMP cases, some mutations were present in more than 30% of the patients, including *SUZ12*, *NOTCH1*, *WT1, IKAF1, PHF6*, *NF1* and *MED12*. While some of those are found at similar frequencies in ETP-ALL, lesions in *NF1* and *MED12* were uniquely abundant in CIMP leukemias. *MED12* is a subunit of the Mediator complex, which plays critical functions in the regulation of transcription at multiple levels, including initiation, pausing and elongation [26]. The Mediator complex has been implicated in short-range interactions in collaboration with cohesin [27,28], but recent studies indicate it may act as a functional rather than an architectural bridge between enhancers and promoters [29,30]. Importantly, MED12 is essential for HSC function by cooperating with p300 in the maintenance of enhancer activity [31]. Together with other mutations affecting epigenetic modifiers, *MED12* lesions can thus contribute to the abnormal epigenetic state of these leukemias. The recurrence of *NF1* copy number losses was also noteworthy. Inactivation of *NF1* results in RAS overactivation and increased blast colony formation and has been associated with poor prognosis in AML [32,33], but is also detected in T-ALL with and without neurofibromatosis [34,35]. The specific contribution of these mutations to the unique epigenetic phenotype of these leukemias remains to be elucidated.

Analysis of motif activity inferred from ATAC-seq pinpoints key dysregulated TFs involved in lineage specification

The regulatory networks driving a differentiation block in CIMP were investigated with *chromVAR*, a tool that estimates TF motif activity by computing bias-corrected deviations in chromatin accessibility at motif-containing peaks relative to the expectation. The most variable motifs across the entire leukemia cohort belonged to the JUN and FOS families of proto-oncogenes, which are positive regulators of myeloid differentiation [36] and are overexpressed in certain AML subtypes [37,38] (Supplementary Figure 8A). Besides, several members of the C/EBP and GATA families were also very variable, consistently with the important

roles in differentiation of genes like *GATA2* [39] or *CEBPA* [40]. As expected, binding sites for CEBP TFs were inaccessible in CIMPs, *CEBPA* DM AML and T-ALL.

Supervised comparisons of motif activity revealed large differences in C/EBP between CIMP and AML, as well as between AML and T-ALL (Figure 5B, Supplementary Figure 8B). This suggests C/EBP activity might be critical for the loss of myeloid potential in CIMP leukemias, in line with previous reports of the essential function of *CEBPA* in establishing the myeloid trajectory [40]. Interestingly, other TFs were significantly more active in CIMP than in T-ALL, including *SPI1* (PU.1), which induces myeloid commitment at high levels[41] and whose downregulation is necessary for terminal T-cell maturation[42], but also BACH1, which promotes B-cell development at the expense of the myeloid lineage [43].

In order to better define the relationship between TF expression and motif activity, we combined *chromVAR* deviations with gene expression of 496 TFs across 79 patients where both ATAC-seq and RNA-seq were available, including 9 CIMP, 51 AML and 19 T-ALL cases. At first glance, the motif activity of the 50 most variable TFs was concordant with their expression, though discrepancies were also apparent (Figure 5C). A possible explanation is the motif similarity of different TFs belonging to the same family, of which only a fraction may be relevant in a given cell type. The loss of accessibility at TFBS of the C/EBP family in CIMP relative to AML was accompanied by transcriptional repression of C/EBP genes, except for CEBPG, which acts as a negative modulator of its homologues (Figure 5C). Conversely, CIMP cases exhibited increased motif activity and expression compared to AML. When comparing CIMP against T-ALL, this integrative analysis revealed loss of motif activity and expression for TCF7, IRF4 and LEF1, whereas BACH2 exhibited gain of TFBS accessibility yet loss of expression. Of note, *LEF1*, which participates in the early stages of thymocyte maturation [44], was among the few genes with promoter hypermethylation in supervised comparisons between CIMP and T-ALL.

In order to disambiguate which TFs are true regulators at their predicted binding sites in leukemia, we calculated the correlation between TFBS accessibility and gene expression, an approach commonly applied to single-cell technologies (Figure 5D, Supplementary Figure 8F, Supplementary Data 34) [45]. This correlation was particularly high for CEBPB and LEF1, which positively regulate the myeloid and the lymphoid program in AML and T-ALL respectively. On the other hand, a negative correlation was observed for KLF16 and ZBTB7B, suggesting they either act as repressors or compete against other members of the same family. Indeed, KLF proteins are known to compete against each other for promoters and enhancers [46], whereas ZBTB7B functions as a repressor, particularly in T-cell differentiation [47]. Furthermore, integration of these correlation coefficients with changes in expression in CIMP revealed dysregulation of key regulators possibly driving their aberrant phenotype (Supplementary Figure 8G). Namely, T-cell regulators like GATA3 or LEF1 were overexpressed in CIMP compared to AML, whereas myeloid regulators such as SPI1, CEBPB or CEBPE were repressed. On the other hand, MEIS1 and HOXA13 were upregulated relative to T-ALL, with other genes like LEF1 and KLF4 exhibiting downregulation. Interestingly, ectopic expression of HOXA13 is associated with an immature phenotype and poor prognosis and is particularly frequent in ETP-ALL [48].

Finally, some of the most variable motifs were evaluated for synergy (Supplementary Figure 8H). CTCF was highly antagonistic with KLF4, CEBPA, SPI1 and FOS, but positively associated with LEF1. Notably, both KLF4 [49,50] and LEF1 [51] are involved in structural reorganization of the genome, like CTCF, with KLF4 forming loops independently of CTCF. CEBPA and FOS were also strongly synergistic, in line with observations that JUN forms heterodimers with CEBPA that direct monocytic differentiation more potently than either of the two TFs alone [52]. However, high JUN expression has also been reported to inhibit CEBPA binding in AML, indicating a possible competitive behaviour as well [53].

Supervised analyses of ATAC-seq and ChIP-seq data identify epigenetic signatures in line with gene expression patterns

To further understand the epigenetic makeup of CIMP leukemias, we conducted additional analyses of the ATAC-seq (Supplementary Data 35) and ChIP-seq (Supplementary Data 36) data mentioned above. Contrary

to methylation and expression data, there were no large differences between CIMP and either AML or T-ALL in terms of both open chromatin (Supplementary Figure 10A) and H3K27ac deposition (Supplementary Figure 10D). Unexpectedly, however, chromatin was slightly more open at promoters in CIMPs relative to AML, despite the widespread hypermethylation leading to gene silencing. Supervised comparisons similarly revealed that even numbers of promoters and enhancers are either active or inactive in CIMP with respect to AML or T-ALL (Supplementary Figures 10B, 10E).

To draw more meaningful conclusions, we conducted GSEA on genes located in the vicinity of these variable peaks, ranked according to their differential binding in each comparison. CIMPs exhibited marked depletion of open chromatin at the targets of the PRC complex, i.e. regions marked by H3K27me3, relative to both AML and CD34+ cells (Supplementary Figure 10C). This is in keeping with the preferential DNA methylation at these same regions, which become increasingly closed as well. Remarkably, CIMPs displayed open chromatin at genes normally expressed in AML when compared to T-ALL, whereas genes involved in T-cell differentiation were closed. Similar observations were derived from GSEA on H3K27ac (Supplementary Figure 10F), but these same T-cell gene sets were more active than in CD34+ cells. Likewise, H3K27ac GSEA in CIMP vs AML revealed upregulation of T-cell sets and downregulation of AML ones. This is consistent with the epigenetic ambiguity of these leukemias and the notion that their differentiation is blocked at an intermediate stage, thus preventing a full commitment to either the T-lymphoid or the myeloid lineages.

Finally, we also generated H3K27me3 ChIP-seq for a few CIMP and AML samples to investigate whether the increases in DNA methylation are accompanied by increased deposition of H3K27me3. Interestingly, there was a slight decrease in global H3K27me3 levels (Supplementary Figure 10G), confirmed by differential analyses with DiffBind (Supplementary Figure 10H). Therefore, the establishment of DNA methylation does not require spreading of H3K27me3 to additional regions. GSEA revealed enrichment of H3K27me3 at genes involved in mitosis and NOTCH signalling and depletion at genes downregulated in various types of AML (*NPM1*-mutant, RUNX1-ETO fusions, etc.). However, none of these results were significant with FDR < 0.05, possibly due to the small size of the dataset.

Additional examples of altered 3D genome structure

In order to detect changes in 3D genome structure that led to alterations in gene expression, we identified variable TADs and loops that contained or overlapped differentially expressed genes. Most of these differences were observed between CIMP and AML, in line with the notion that CIMP leukemias may derive from a lymphoid progenitor.

We first investigated whether any TFs silenced by promoter methylation exhibited concomitant changes in chromatin interactions. Only three of these TFs were found in loops with decreased intensity in the CIMP group: *PKNOX2*, *KLF4* (Figure 7G) and *CEBPD* (Figure 7H). In the latter two, the loss of interaction was accompanied by reduced CTCF binding and gain of methylation at the *CEBPD* and *KLF4* promoters. On the other hand, 7 downregulated genes were found in ΔTADs, including again *CEBPD* and *KLF4,* but also other TFs like *TAL1*, *IRF8* (Supplementary Figure 12F) and *MAFB*. In view of these observations, changes in 3D structure may contribute to silencing of TFs driving a differentiation block, but they are dispensable for a process that is largely driven by promoter silencing.

Next, we conducted an unbiased survey of ΔTADs (Supplementary Data 40) and DIs (Supplementary Data 41) with associated changes in CTCF binding and potential implications for gene expression. Aside from the examples described in the main text, the *IRF8* TAD exhibits reduced insulation accompanied by a loss of CTCF binding on the right boundary. Similarly, the TAD containing *GASK1B* was partially lost, potentially leading to downregulation of this gene due to the lack of interaction with a proximal enhancer (Supplementary Figure 12G). On the other hand, the *ANGPT2* TAD became strongly insulated, possibly resulting in upregulation of this gene (Supplementary Figure 12H). Gain of interaction between *GATA3* and a putative enhancer that is only active in CIMP and not in AML possibly leads to overexpression of *GATA3* (Figure 7I). Likewise, a loop involving the promoters of *DNMT3B* (Supplementary Figure 12I) and *DNAJC1* (Supplementary Figure 12J) are

also acquired in CIMP cases, possibly leading to overexpression of these genes. In contrast, the loss of interaction between *IL6* and putative upstream enhancer elements may explain the downregulation of this gene, whose promoter was unmethylated (Supplementary Figure 12K).

There were only 3 ΔTADs and DIs between CIMP and T-ALL with reduced CTCF binding, one of which was the loss of insulation at the TAD containing *ASCC1* (Supplementary Figure 12L). This limited chromatin remodelling is in keeping with the notion that these leukemias originate from a lymphoid-biased cell. On the other hand, there were also fewer T-ALL replicates, which decreased the statistical power to detect such changes. The 8 differential enhancer-promoter loops between CIMP and T-ALL did not exhibit a clear correlation with gene expression (Figure 7F). Among those DIs was a loss of interaction between the promoter of the longer *DIPK1A* isoforms and downstream exonic regions of the same gene, which is downregulated in CIMPs (Supplementary Figure 12M). This downregulation is accompanied by a loss of H3K27ac at said promoter. Another example is the gained interaction between *RCC2* and distal regulatory elements close to *SDHB*, which could contribute to the overexpression of that gene (Supplementary Figure 12N).

Loss of *CEBPA* plays a critical role in shaping the leukemic epigenome

Among the TFs downregulated by methylation was *CEBPA*, the loss of which was originally identified as the defining feature of the CIMP-EMC cohort [54], also known as *CEBPA*-silenced. In line with the initial reports, a recurrent observation across the analyses of epigenomics data was that CIMP leukemias exhibited a profound similarity with double mutant AML (Supplementary Figures 2A-G). Double *CEBPA* mutations define an AML subtype (CEBPA DM) with a distinct gene expression profile, comparable to that of CIMP leukemias[54,55]. These patients typically exhibit a combination of N- and C-terminal mutations in the CEBPA protein that disrupt its normal function [56]. Moreover, CIMPs also clustered in the vicinity of AMLs with t(8;21), a chromosomal aberration that produces a RUNX1-RUNXT1 fusion protein, which inhibits the expression of *CEBPA* [57]. The similarity between epigenetic profiles of *CEBPA* DM AMLs and CIMP suggests that the loss of function of *CEBPA*, either by genetic or epigenetic hits, drives the acquisition of a distinct epigenetic and transcriptional landscape.

Furthermore, analysis of motif activity using *chromVAR* revealed that CEBPA and other members of the C/EBP family were among the top 30 TFs with the largest variability in chromatin accessibility across the whole cohort (Supplementary Figure 8A). Importantly, C/EBP TFs were among the few with a significant loss of activity in CIMP relative to AML whereas they displayed the largest increases of accessibility in AML compared to T-ALL (Figure 5B). Integration of gene expression data further confirmed that C/EBP family members are the only TFs showing simultaneous loss of expression and TFBS accessibility at significant levels (Figure 5E). Similarly, TF footprinting with *TOBIAS* identified various members of the C/EBP family as unbound in the CIMP group relative to AML (Supplementary Figure 8C).

Altogether, this underscores the importance of *CEBPA* as a critical determinant of cell identity and supports the notion that its loss in CIMP leukemias underlies their unique differentiation status. Of note, the +42-kb hematopoietic enhancer of *CEBPA* [58] is active in both CIMP and AML, but not in T-ALL (Supplementary Figure 13A-B). Therefore, it can be surmised that transformation takes place in a cell type that would normally be primed to express *CEBPA*, and thus exhibits some degree of multilineage priming.

SUPPLEMENTARY REFERENCES

1. Touzart, A. *et al.* Epigenetic analysis of patients with T-ALL identifies poor outcomes and a hypomethylating agent-responsive subgroup. *Sci. Transl. Med.* **13**, (2021).
2. Zhang, J. *et al.* The genetic basis of early T-cell precursor acute lymphoblastic leukaemia. *Nature* **481**, 157–163 (2012).
3. Xie, X. *et al.* Single-cell transcriptomic landscape of human blood cells. *Natl. Sci. Rev.* **8**, (2021).
4. Laurenti, E. *et al.* The transcriptional architecture of early human hematopoiesis identifies multilevel control of lymphoid commitment. *Nat. Immunol.* **14**, 756–763 (2013).
5. Martens, J. H. A. & Stunnenberg, H. G. BLUEPRINT: mapping human blood cell epigenomes. *Haematologica* **98**, 1487–9 (2013).
6. ENCODE Project Consortium. An integrated encyclopedia of DNA elements in the human genome. *Nature* **489**, 57–74 (2012).
7. Speir, M. L. *et al.* The UCSC Genome Browser database: 2016 update. *Nucleic Acids Res.* **44**, D717-25 (2016).
8. Sandelin, A., Alkema, W., Engström, P., Wasserman, W. W. & Lenhard, B. JASPAR: an open-access database for eukaryotic transcription factor binding profiles. *Nucleic Acids Res.* **32**, D91-4 (2004).
9. Tyner, J. W. *et al.* Functional genomic landscape of acute myeloid leukaemia. *Nature* **562**, 526–531 (2018).
10. The Cancer Genome Atlas Research Network *et al.* Genomic and epigenomic landscapes of adult de novo acute myeloid leukemia. *N. Engl. J. Med.* **368**, 2059–74 (2013).
11. Kalender Atak, Z. *et al.* Comprehensive Analysis of Transcriptome Variation Uncovers Known and Novel Driver Events in T-Cell Acute Lymphoblastic Leukemia. *PLoS Genet.* **9**, e1003997 (2013).
12. Liu, Y. *et al.* The genomic landscape of pediatric and young adult T-lineage acute lymphoblastic leukemia. *Nat. Genet.* **49**, 1211–1218 (2017).
13. Chen, B. *et al.* Identification of fusion genes and characterization of transcriptome features in T-cell acute lymphoblastic leukemia. *Proc. Natl. Acad. Sci. U. S. A.* **115**, 373–378 (2017).
14. Neumann, M. *et al.* Mutational spectrum of adult T-ALL. *Oncotarget* **6**, 2754–2766 (2015).
15. Landt, S. G. *et al.* ChIP-seq guidelines and practices of the ENCODE and modENCODE consortia. *Genome Res.* **22**, 1813–31 (2012).
16. Neumann, M. *et al.* Whole-exome sequencing in adult ETP-ALL reveals a high rate of DNMT3A mutations. *Blood* **121**, 4749–4752 (2013).
17. Alexander, T. B. *et al.* The genetic basis and cell of origin of mixed phenotype acute leukaemia. *Nature* **562**, 373–406 (2018).
18. Xiao, W. *et al.* PHF6 and DNMT3A mutations are enriched in distinct subgroups of mixed phenotype acute leukemia with T-lineage differentiation. *Blood Adv.* **2**, 3526–3539 (2018).
19. Yang, F. *et al.* Identification and prioritization of myeloid malignancy germline variants in a large cohort of adult patients with AML. *Blood* **139**, 1208–1221 (2022).
20. Janiszewska, H. *et al.* Constitutional mutations of the CHEK2 gene are a risk factor for MDS, but not for de novo AML. *Leuk. Res.* **70**, 74–78 (2018).
21. Zhang, S. J. *et al.* Gain-of-function mutation of GATA-2 in acute myeloid transformation of chronic myeloid leukemia. *Proc. Natl. Acad. Sci. U. S. A.* **105**, 2076–2081 (2008).
22. Churpek, J. E. *et al.* Inherited mutations in cancer susceptibility genes are common among survivors of breast cancer who develop therapy-related leukemia. *Cancer* **122**, 304–311 (2016).
23. Simonetti, G. *et al.* Aneuploid acute myeloid leukemia exhibits a signature of genomic alterations in the cell cycle and protein degradation machinery. *Cancer* **125**, 712–725 (2019).
24. Mrózek, K., Heerema, N. A. & Bloomfield, C. D. Cytogenetics in acute leukemia. *Blood Rev.* **18**, 115–136 (2004).
25. Walter, M. J. *et al.* Acquired copy number alterations in adult acute myeloid leukemia genomes. *Proc. Natl. Acad. Sci. U. S. A.* **106**, 12950–12955 (2009).
26. Allen, B. L. & Taatjes, D. J. The Mediator complex: A central integrator of transcription. *Nature Reviews Molecular Cell Biology* vol. 16 155–166 at https://doi.org/10.1038/nrm3951 (2015).
27. Phillips-Cremins, J. E. *et al.* Architectural protein subclasses shape 3D organization of genomes during lineage commitment. *Cell* **153**, 1281–1295 (2013).
28. Kagey, M. H. *et al.* Mediator and cohesin connect gene expression and chromatin architecture. *Nature* **467**, 430–435 (2010).
29. El Khattabi, L. *et al.* A Pliable Mediator Acts as a Functional Rather Than an Architectural Bridge between Promoters and Enhancers. *Cell* **178**, 1145-1158.e20 (2019).
30. Jaeger, M. G. *et al.* Selective Mediator dependence of cell-type-specifying transcription. *Nat. Genet.* **52**, 719–727 (2020).

31. Aranda-Orgilles, B. *et al.* MED12 Regulates HSC-Specific Enhancers Independently of Mediator Kinase Activity to Control Hematopoiesis. *Cell Stem Cell* **19**, 784–799 (2016).

32. Parkin, B. *et al.* NF1 inactivation in adult acute myelogenous leukemia. *Clin. Cancer Res.* **16**, 4135–4147 (2010).

33. Eisfeld, A. K. *et al.* NF1 mutations are recurrent in adult acute myeloid leukemia and confer poor outcome. *Leukemia* **32**, 2536–2545 (2018).

34. Balgobind, B. V. *et al.* Leukemia-associated NF1 inactivation in patients with pediatric T-ALL and AML lacking evidence for neurofibromatosis. *Blood* **111**, 4322–4328 (2008).

35. Galbiati, M. *et al.* Natural history of acute lymphoblastic leukemia in neurofibromatosis type 1 monozygotic twins. *Leukemia* vol. 27 1778–1781 at https://doi.org/10.1038/leu.2013.55 (2013).

36. Lord, K. A., Abdollahi, A., Hoffman-Liebermann, B. & Liebermann, D. A. Proto-oncogenes of the fos/jun family of transcription factors are positive regulators of myeloid differentiation. *Mol. Cell. Biol.* **13**, 841–851 (1993).

37. Elsässer, A. *et al.* The fusion protein AML1-ETO in acute myeloid leukemia with translocation t(8;21) induces c-jun protein expression via the proximal AP-1 site of the c-jun promoter in an indirect, JNK-dependent manner. *Oncogene* **22**, 5646–5657 (2003).

38. Staber, P. B. *et al.* Common alterations in gene expression and increased proliferation in recurrent acute myeloid leukemia. *Oncogene* **23**, 894–904 (2004).

39. Rodrigues, N. P. *et al.* GATA-2 regulates granulocyte-macrophage progenitor cell function. *Blood* **112**, 4862–4873 (2008).

40. Zhang, P. *et al.* Enhancement of hematopoietic stem cell repopulating capacity and self-renewal in the absence of the transcription factor C/EBPα. *Immunity* **21**, 853–863 (2004).

41. Nerlov, C. & Graf, T. PU.1 induces myeloid lineage commitment in multipotent hematopoietic progenitors. *Genes Dev.* **12**, 2403–2412 (1998).

42. Anderson, M. K., Weiss, A. H., Hernandez-Hoyos, G., Dionne, C. J. & Rothenberg, E. V. Constitutive expression of PU.1 in fetal hematopoietic progenitors blocks T cell development at the pro-T cell stage. *Immunity* **16**, 285–296 (2002).

43. Itoh-Nakadai, A. *et al.* The transcription repressors Bach2 and Bach1 promote B cell development by repressing the myeloid program. *Nat. Immunol.* **15**, 1171–1180 (2014).

44. Okamura, R. M. *et al.* Redundant regulation of T cell differentiation and TCRα gene expression by the transcription factors LEF-1 and TCF-1. *Immunity* **8**, 11–20 (1998).

45. Buenrostro, J. D. *et al.* Integrated Single-Cell Analysis Maps the Continuous Regulatory Landscape of Human Hematopoietic Differentiation. *Cell* **173**, 1535-1548.e16 (2018).

46. Ilsley, M. D. *et al.* Krüppel-like factors compete for promoters and enhancers to fine-tune transcription. *Nucleic Acids Res.* **45**, 6572–6588 (2017).

47. Wang, L. *et al.* The Zinc Finger Transcription Factor Zbtb7b Represses CD8-Lineage Gene Expression in Peripheral CD4+ T Cells. *Immunity* **29**, 876–887 (2008).

48. Yang, L. *et al.* 3D genome alterations associated with dysregulated HOXA13 expression in high-risk T-lineage acute lymphoblastic leukemia. *Nat. Commun.* **12**, 1–12 (2021).

49. Di Giammartino, D. C. *et al.* KLF4 is involved in the organization and regulation of pluripotency-associated three-dimensional enhancer networks. *Nat. Cell Biol.* **21**, 1179–1190 (2019).

50. Wei, Z. *et al.* Klf4 organizes long-range chromosomal interactions with the OCT4 locus inreprogramming andpluripotency. *Cell Stem Cell* **13**, 36–47 (2013).

51. Shan, Q. *et al.* Tcf1 and Lef1 provide constant supervision to mature CD8+ T cell identity and function by organizing genomic architecture. *Nat. Commun.* **12**, 1–20 (2021).

52. Cai, D. H. *et al.* C/EBPα:AP-1 leucine zipper heterodimers bind novel DNA elements, activate the PU.1 promoter and direct monocyte lineage commitment more potently than C/EBPα homodimers or AP-1. *Oncogene* **27**, 2772–2779 (2008).

53. Rangatia, J. *et al.* Elevated c-Jun expression in acute myeloid leukemias inhibits C/EBPα DNA binding via leucine zipper domain interaction. *Oncogene* **22**, 4760–4764 (2003).

54. Wouters, B. J. *et al.* Distinct gene expression profiles of acute myeloid/T-lymphoid leukemia with silenced CEBPA and mutations in NOTCH1. *Blood* **110**, 3706–3714 (2007).

55. Wouters, B. J. *et al.* Double CEBPA mutations, but not single CEBPA mutations, define a subgroup of acute myeloid leukemia with a distinctive gene expression profile that is uniquely associated with a favorable outcome. *Blood* **113**, 3088–3091 (2009).

56. Fasan, A. *et al.* The role of different genetic subtypes of CEBPA mutated AML. *Leukemia* **28**, 794–803 (2014).

57. Pabst, T. *et al.* AML1-ETO downregulates the granulocytic differentiation factor C/EBPα in t(8;21) myeloid leukemia. *Nat. Med.* **7**, 444–451 (2001).

58. Avellino, R. *et al.* An autonomous CEBPA enhancer specific for myeloid-lineage priming and neutrophilic differentiation. *Blood* **127**, 2991–3003 (2016).

59. Subramanian, A. *et al.* Gene set enrichment analysis: A knowledge-based approach for interpreting genome-wide

expression profiles. *Proc. Natl. Acad. Sci. U. S. A.* **102**, 15545–15550 (2005).

60. Sergushichev, A. A. An algorithm for fast preranked gene set enrichment analysis using cumulative statistic calculation. *bioRxiv* 060012 (2016) doi:10.1101/060012.

61. Yu, G., Wang, L. G., Han, Y. & He, Q. Y. ClusterProfiler: An R package for comparing biological themes among gene clusters. *Omi. A J. Integr. Biol.* **16**, 284–287 (2012).

62. Love, M. I., Huber, W. & Anders, S. Moderated estimation of fold change and dispersion for RNA-seq data with DESeq2. *Genome Biol.* **15**, 550 (2014).

63. Ross-Innes, C. S. *et al.* Differential oestrogen receptor binding is associated with clinical outcome in breast cancer. *Nature* **481**, 389–393 (2012).
