# Supplementary material for: Epigenetic alterations affecting hematopoietic regulatory networks as drivers of mixed myeloid/lymphoid leukemia
Source: Nat Commun. 2024 Jul 7;15:5693. doi: 10.1038/s41467-024-49811-y (PMC11228033; doi:10.1038/s41467-024-49811-y)
Supplement: Supplementary file 3 — Description of Additional Supplementary Files [file 41467_2024_49811_MOESM3_ESM.pdf]

DESCRIPTION OF ADDITIONAL SUPPLEMENTARY FILES

SUPPLEMENTARY DATA

The Supplementary Data are included as separate tabs in a single Microsoft Excel file.

Dataset name: Supplementary Data 1.
Description: Summary of data generated for each patient.

Dataset name: Supplementary Data 2.
Description: Point mutations and small indels identified in CIMP leukemias based on WES data.

Dataset name: Supplementary Data 3.
Description: Copy number alterations (CNAs) detected by CNVkit in WES data from CIMP leukemias.

Dataset name: Supplementary Data 4.
Description: Number and type of CNAs in protein-coding genes, related to Supplementary Data 3.

Dataset name: Supplementary Data 5
Description: Fusion genes identified in RNA-seq data from CIMP leukemias using an ensemble of tools.

Dataset name: Supplementary Data 6.
Description: Comparison between the frequencies of recurrent point mutations and indels in CIMP and ETP-ALL, T/M MPAL, T-ALL and AML, according to previous publications (split by cohort).

Dataset name: Supplementary Data 7.
Description: Comparison between the frequencies of recurrent point mutations and indels in CIMP and ETP-ALL, T/M MPAL, T-ALL and AML, according to previous publications (summary). The association (or lack thereof) between CIMP and genetic lesions was investigated with a two-sided Fisher's exact test in pairwise comparisons with other leukemias.

Dataset name: Supplementary Data 8.
Description: Comparison between the frequencies of recurrent gene fusions in CIMP and ETP-ALL, T/M MPAL, T-ALL and AML, according to previous publications.

Dataset name: Supplementary Data 9.
Description: Differential gene expression in CIMP relative to AML, T-ALL and CD34+ cells. The statistical significance was determined by a two-sided Wald test in the DESeq2 package (pvalue columns) and adjusted for multiple testing with the Benjamini–Hochberg procedure (padj columns).

Dataset name: Supplementary Data 10.
Description: Results from GSEA on RNA-seq comparing CIMP vs AML using a customized C2 gene set. The nominal p-values (pval) were calculated by a permutation test (n=10000) using the *fgsea* R package. As described in [59,60], a test statistic is calculated for each permutation of the data; the p-value is the number of times this statistic is above the value of the test statistic in the original data, divided by the number of permutations. Adjusted p-values were calculated by the Benjamini–Hochberg procedure.

Dataset name: Supplementary Data 11.
Description: Results from GSEA on RNA-seq comparing CIMP vs T-ALL using a customized C2 gene set. Statistical methodology was conducted as described for **Supplementary Data 11**.

Dataset name: Supplementary Data 12.
Description: Results from GSEA on RNA-seq comparing CIMP vs CD34+ cells using a customized C2 gene set. Statistical methodology was conducted as described for **Supplementary Data 11**.

Dataset name: Supplementary Data 13.
Description: Differentially methylated regions (DMR) at peaks identified by MCIP-seq. Statistical significance was determined by a two-sided Wald test in the DESeq2 package (pvalue columns) and adjusted for multiple testing with the Benjamini–Hochberg procedure (padj columns).

Dataset name: Supplementary Data 14.
Description: Differentially methylated regions (DMR) calculated from MethylationEPIC array data between CIMP and AML. Statistical methodology was conducted as described for **Supplementary Data 13.**

Dataset name: Supplementary Data 15.
Description: Differentially methylated regions (DMR) calculated from MethylationEPIC array data between CIMP and T-ALL. Statistical methodology was conducted as described for **Supplementary Data 13**.

Dataset name: Supplementary Data 16.
Description: Differentially methylated regions (DMR) calculated from MethylationEPIC array data between CIMP and ETP-ALL. Statistical methodology was conducted as described for **Supplementary Data 13.**

Dataset name: Supplementary Data 17.
Description: Results from GO term enrichment performed on MethylationEPIC array DMRs between CIMP and AML. Statistical significance was determine by a hypergeometric test with the ClusterProfiler R package [61]. Adjusted p-values were calculated by a) the Benjamini–Hochberg procedure implemented in the p.adjust R package (p.adjust column), and b) the false discovery rate (FDR) estimation procedure implemented in the qvalue package (qvalue column).

Dataset name: Supplementary Data 18.
Description: Results from GO term enrichment performed on MethylationEPIC array DMRs between CIMP and T-ALL. Statistical methodology was conducted as described for **Supplementary Data 17.**

Dataset name: Supplementary Data 19.
Description: Results from GO term enrichment performed on MethylationEPIC array DMRs between CIMP and ETP-ALL. Statistical methodology was conducted as described for **Supplementary Data 17.**

Dataset name: Supplementary Data 20.
Description: Results from GSEA on MCIP-seq comparing CIMP vs AML (C2 and C5 collections). Statistical methodology was conducted as described for **Supplementary Data 17**.

Dataset name: Supplementary Data 21.
Description: Results from GSEA on MCIP-seq comparing CIMP vs T-ALL (C2 and C5 collections). Statistical methodology was conducted as described for **Supplementary Data 17**.

Dataset name: Supplementary Data 22.
Description: Results from GSEA on MCIP-seq comparing CIMP vs CD34+ cells (C2 and C5 collections). Statistical methodology was conducted as described for **Supplementary Data 17.**

Dataset name: Supplementary Data 23.
Description: Integration of differential methylation and expression at gene promoters.

Dataset name: Supplementary Data 24.
Description: Integration of differential gene expression and chromatin accessibility at enhancers.

Dataset name: Supplementary Data 25.
Description: Integration of differential gene expression and H3K27ac signal at enhancers.

Dataset name: Supplementary Data 26.
Description: Results from locus overlap analysis with LOLA of MethylationEPIC array data DMRs between CIMP and AML. The statistical significance of the overlap between DMRs and ChIP datasets was determined with a one-sided Fisher's exact test, and the result p-value was transformed with -log10 (pValueLog). The column rnkPV indicates the p-value rank for each dataset in the database. Adjusted p-values were calculated by the false discovery rate (FDR) estimation procedure implemented in the qvalue package (qvalue column).

Dataset name: Supplementary Data 27.
Description: Results from locus overlap analysis with LOLA of MethylationEPIC array data DMRs between CIMP and T-ALL. Statistical methodology was conducted as described for **Supplementary Data 26**.

Dataset name: Supplementary Data 28.
Description: Results from locus overlap analysis with LOLA of MethylationEPIC array data DMRs between CIMP and ETP-ALL. Statistical methodology was conducted as described for **Supplementary Data 26**.

Dataset name: Supplementary Data 29.
Description: Results from locus overlap analysis with LOLA of MCIP-seq data DMRs between CIMP and AML. Statistical methodology was conducted as described for **Supplementary Data 26**.

Dataset name: Supplementary Data 30.
Description: Results from locus overlap analysis with LOLA of MCIP-seq data DMRs between CIMP and T-ALL. Statistical methodology was conducted as described for **Supplementary Data 26**.

Dataset name: Supplementary Data 31.
Description: Differential motif activity measured by chromVAR in ATAC-seq data. The statistical significance of the difference (diff column) in chromosomal accessibility between groups at a given motif was determined by a two-sided Wilcoxon test (pvalue column). Adjusted p-values were calculated by the Benjamini–Hochberg procedure implemented in the p.adjust package (FDR column).

Dataset name: Supplementary Data 32.
Description: Results of TF footprinting analysis conducted with TOBIAS in CIMP vs AML ATAC-seq data.

Dataset name: Supplementary Data 33.
Description: Results of TF footprinting analysis conducted with TOBIAS in CIMP vs T-ALL ATAC-seq data.

Dataset name: Supplementary Data 34.
Description: Correlation between motif activity and gene expression for transcription factors.

Dataset name: Supplementary Data 35.
Description: Differential chromatin accessibility between CIMP and AML, T-ALL or CD34+ cells**.** The statistical significance of pairwise comparisons at every peak was determined by a two-sided Wald test

(p.value columns) and adjusted for multiple testing with the Benjamini–Hochberg procedure (FDR columns). These operations were performed with the help of the DESeq2 [62] and DiffBind [63] R packages.

Dataset name: Supplementary Data 36.
Description: Differential H3K27ac signal between CIMP and AML, T-ALL or CD34+ cells. Statistical analyses were conducted as described in the legend of **Supplementary Data 35.**

Dataset name: Supplementary Data 37**.**
Description: Differential H3K27me3 signal between CIMP and AML. Statistical analyses were conducted as described in the legend of **Supplementary Data 35.**

Dataset name: Supplementary Data 38.
Description: Consensus list of CTCF binding sites and comparisons between CIMP and AML or T-ALL**.** Statistical analyses were conducted as described in the legend of **Supplementary Data 35**.

Dataset name: Supplementary Data 39.
Description: Integration of differential methylation and CTCF binding analyses. Statistical analyses for both DNA methylation and CTCF binding were conducted as described in the legend of **Supplementary Data 35**.

Dataset name: Supplementary Data 40.
Description: Differential TAD insulation between leukemias, including genes with differential expression within the TAD and change of CTCF binding at the boundaries. The statistical significance of pairwise comparisons at every region was determined by a two-sided Wald test (p.value columns) and adjusted for multiple testing with the Benjamini–Hochberg procedure (padj columns). In all cases, the calculation was done with DESeq2, but the input depended on the data source: TAD insulation was derived from Hi-C data using the HOMER suite, gene expression was obtained from RNA-seq data with Salmon and CTCF binding was quantified and normalized with DiffBind. Only the CTCF binding site with the most significant change (lowest FDR) was selected for each boundary.

Dataset name: Supplementary Data 41.
Description: Differential loops between leukemias, including information about enhancers and promoters in the proximity of the anchors. Statistical analyses were conducted as described in the legend of Supplementary Data 40. Here, however, loop score was derived from Hi-C with HOMER and used to calculate differential interactions with DESeq2.

SUPPLEMENTARY CODE 1

The Supplementary Code is provided as a single ZIP file entitled that contains all the scripts used in the analysis of the data presented in this manuscript. A brief description of each is provided below:

Script name: atac_analysis.R
Description: Motif footprinting and differential variability analyses of ATAC-seq data

Script name: chiptf_analysis.R
Description: Analyses of TCF7, SPI1 and CEBPA ChIP-seq data, using the ComplexHeatmap and DiffBind packages

Script name: cibersort_analysis.R

Script name: Preparation of the reference and mixture matrices for analyses with CIBERSORTx, as well as functions for plotting the results

Script name: CIMP_CTCF_Effect.R
Description: Analyses of CTCF ChIP-seq data, including differential occupancy with DiffBind, motif analyses and quantification of GC content at each peak.

Script name: CIMP_epigenetics_clustering.R
Description: Dimensionality reduction and clustering of various epigenetics data and transcriptomics, namely MCIP-seq, RNA-seq, ATAC-seq and H3K27ac ChIP-seq

Script name: CIMP_mcip_rnaseq3.R
Description: Integrative analysis of RNA-seq and MCIP-seq data.

Script name: cimp_project_functions.R
Description: Utility functions used in other scripts related to this research.

Script name: CIMP_signature_analysis.R
Description: Transcriptional signature analyses with GSEA and single sample GSEA.

Script name: epic_array.R
Description: Analysis of Infinium MethylationEPIC array data.

Script name: epigenetic_integration.R
Description: This script contains 1) functions for assignment of putative enhancers to their cognate promoters, 2) code to generate plots that compare the activity of promoters by H3K27ac or ATAC-seq data with the expression of their assigned promoters.

Script name: get_jaspar_matrices.R
Description: Code used to obtain information missing from the position weight matrix (PWM) TXT file available at the JASPAR website. First, it downloads the metadata of all human transcription factors (TF) in JSON format, including type, class, family and name. After converting them to a suitable format, the script uses this information to fill the "tags" slot for each TF in the TXT file.

Script name: hic_data_analysis2.R
Description: Analysis of Hi-C data previously processed with HOMER. The script uses DESeq2 to normalize the data, create a PCA plot and calculate differential loops and TADs.

Script name: histone_marks_analysis.R
Description: Differential accessibility/binding analyses of ATAC-seq, H3K27ac ChIP-seq and H3K27me3 ChIP-seq shown in Supplementary Figure 10.

Script name: MCIP_analysis.R
Description: Analysis of MCIP-seq data, including differential methylation and quantification of methylation at various genomic features.

Script name: metanalysis_ETP_MPAL.R
Description: Comparison of mutational frequencies in CIMP leukemias and other acute leukemias.

Script name: plot_cnv_cimp.R
Description: Generation of an oncoprint with mutational data of CIMP leukemias.
