# Supplementary material for: Epigenetic alterations affecting hematopoietic regulatory networks as drivers of mixed myeloid/lymphoid leukemia
Source: Nat Commun. 2024 Jul 7;15:5693. doi: 10.1038/s41467-024-49811-y (PMC11228033; doi:10.1038/s41467-024-49811-y)
Supplement: Supplementary file 6 — Reporting Summary [file 41467_2024_49811_MOESM6_ESM.pdf]

nature portfolio

Corresponding author(s): Ruud Delwel

Last updated by author(s): Aug 21, 2023

Reporting Summary

Nature Portfolio wishes to improve the reproducibility of the work that we publish. This form provides structure for consistency and transparency in reporting. For further information on Nature Portfolio policies, see our Editorial Policies and the Editorial Policy Checklist.

Statistics

For all statistical analyses, confirm that the following items are present in the figure legend, table legend, main text, or Methods section.

n/a	Confirmed	
☐	☒	The exact sample size (*n*) for each experimental group/condition, given as a discrete number and unit of measurement
☐	☒	A statement on whether measurements were taken from distinct samples or whether the same sample was measured repeatedly
☐	☒	The statistical test(s) used AND whether they are one- or two-sided<br>*Only common tests should be described solely by name; describe more complex techniques in the Methods section.*
☒	☐	A description of all covariates tested
☐	☒	A description of any assumptions or corrections, such as tests of normality and adjustment for multiple comparisons
☐	☒	A full description of the statistical parameters including central tendency (e.g. means) or other basic estimates (e.g. regression coefficient) AND variation (e.g. standard deviation) or associated estimates of uncertainty (e.g. confidence intervals)
☐	☒	For null hypothesis testing, the test statistic (e.g. *F*, *t*, *r*) with confidence intervals, effect sizes, degrees of freedom and *P* value noted<br>*Give P values as exact values whenever suitable.*
☒	☐	For Bayesian analysis, information on the choice of priors and Markov chain Monte Carlo settings
☒	☐	For hierarchical and complex designs, identification of the appropriate level for tests and full reporting of outcomes
☐	☒	Estimates of effect sizes (e.g. Cohen's *d*, Pearson's *r*), indicating how they were calculated

Our web collection on statistics for biologists contains articles on many of the points above.

Software and code

Policy information about availability of computer code

Data collection	No software was used for collection of data.
Data analysis	- MCIP-seq: MCIP-seq reads were aligned to the human reference genome build hg19 with bowtie (v1.1.1) and bigwig files were generated for visualization with deepTools bamCoverage (v3.5.1) and the options --normalizeUsing RPKM --smoothLength 100 --binSize 20. Peak calling was performed with MACS2 (2.2.7.1) using default settings and input DNA as a control. The resulting peaks were filtered against the ENCODE blacklisted regions. Furthermore, a list of regions accessible by MCIP-seq was defined based on data from monocytes treated with the CpG Methyltransferase SssI. All peaks not overlapping with this list were discarded using bedtools intersect.<br><br>- RNA-seq: Salmon v0.13.1 was used to quantify expression of individual transcripts, which were subsequently aggregated to estimate gene-level abundances with tximport. Both gene- and transcript-level abundances were normalized to counts per million (CPM) for visualization in the figures of this paper. Differential gene expression analysis of count estimates from Salmon was performed with DEseq2.<br><br>- ChIP-seq: ChIP-seq reads were aligned to the human reference genome build hg19 with either bowtie (v1.1.1) for single-end data or bowtie2 (v2.3.4.1) for paired-end data. Bigwig files were generated for visualization as described for MCIP-seq. For data with narrow read distributions (H3K27ac, CTCF, PU.1, CEBPA, TCF7), peak calling was performed with MACS2 (v 2.2.7.1) using default settings and the resulting peaks were filtered against the ENCODE blacklisted regions. For H3K27me3, which is found in broad domains, peak calling was performed with EPIC2.<br><br>- ATAC-seq: ATAC-seq reads were aligned to the human reference genome build hg19 with bowtie2 v2.3.4.1, allowing for a maximum 2000 bp insert size. Mitochondrial reads and fragments with mapping quality below 10 were removed. Bigwig files were generated as described for ChIP-seq.

- HiC: Hi-C reads were first processed with HiCUP 120 v0.8.2, which included alignment to hg19 with bowtie2 (v2.3.4.1). Filtered di-tags were then extracted with the script hicup2juicer and subsequently binned with juicer tools pre 121 v1.22.01 at the default resolutions. The resulting .hic files were used for visualization. TADs and loops were identified for each group of leukemias with the findTADsAndLoops.pl find script of the HOMER suite with the parameters –res 5000 and –window 10000.

- Others: Statistical tests were conducted on R version 4.1.0 unless otherwise specified. Most plots were generated using the ggplot2 R package, whereas heatmaps were created with ComplexHeatmap and genomic regions were visualized with plotgardener.

For manuscripts utilizing custom algorithms or software that are central to the research but not yet described in published literature, software must be made available to editors and reviewers. We strongly encourage code deposition in a community repository (e.g. GitHub). See the Nature Portfolio guidelines for submitting code & software for further information.

Data

Policy information about availability of data

All manuscripts must include a data availability statement. This statement should provide the following information, where applicable:

- Accession codes, unique identifiers, or web links for publicly available datasets
- A description of any restrictions on data availability
- For clinical datasets or third party data, please ensure that the statement adheres to our policy

The raw RNA-seq data of AML patients have been previously used in another study 153 and are available at the European Genome-phenome Archive (EGA) [https://ega-archive.org] under accession number EGAS00001004684. All the other raw sequencing data derived from donors or patients have been generated in this study and are deposited at the EGA under accession number EGAS00001007094. This EGA study includes the following datasets: MCIP-seq, RNA-seq, ATAC-seq, ChIP-seq (H3K27ac, CTCF, SPI1, CEBPA, TCF7) and Hi-C. Since these data are derived from human subjects, they are only available under restricted access, which can be requested for each dataset separately on the EGA website. Requestors must sign a data access agreement outlining the terms and conditions for data use and fill in a form specifying their research question. Requests will be processed within 1 week, and the data will be available for a maximum of 2 years unless an appeal for extension is submitted.

Processed data are publicly available in ArrayExpress [https://www.ebi.ac.uk/biostudies/arrayexpress] with the following identifiers: E-MTAB-13117 (CTCF ChIP-seq), E-MTAB-13118 (ATAC-seq), E-MTAB-13119 (H3K27ac ChIP-seq), E-MTAB-13120 (MCIP-seq), E-MTAB-13121 (RNA-seq), E-MTAB-13122 (Hi-C), E-MTAB-14060 (TF ChIP-seq)

In addition, we have used publicly available data from the ENCODE [http://www.encodeproject.org/] and FANTOM [https://fantom.gsc.riken.jp/5/] consortia, as well as a single-cell RNA-seq dataset of hematopoietic cells obtained from the Gene Expression Omnibus (GEO) [https://www.ncbi.nlm.nih.gov/geo/] database under the accession code GSE149938. We also used Illumina Infinium MethylationEPIC data from T-ALL (GSE147667) and AML (GSE159907) available at GEO.

Research involving human participants, their data, or biological material

Policy information about studies with human participants or human data. See also policy information about sex, gender (identity/presentation), and sexual orientation and race, ethnicity and racism.

Reporting on sex and gender	Sex and gender were not considered in the study design, that is, samples were collected and analyzed without considering their sex or gender. However, sex information was still collected on a self-reported basis and used to confirm there was no systematic bias that could confound the results. We also inferred the sex from cytogenetics and NGS data.
Reporting on race, ethnicity, or other socially relevant groupings	No socially relevant categorization data, such as race or ethnicity, were collected in this study.
Population characteristics	The study was focused on patients diagnosed with acute myeloid leukemia (AML) that exhibited a CpG Island methylator phenotype (CIMP) and mixed lymphoid/myeloid surface markers. To compare them to other leukemias, we also retrieved data from non-CIMP AML patients and T-ALL cases. The selection of those patients was largely random, based primarily on the availability of biological material. The patients included in the study had ages ranging from 15 to 87 at diagnosis, with an average of 48. Where available, the age of the patients is indicated in Supplementary Table 1, approximated to the closest decade.
Recruitment	Samples of AML, CIMP, T-ALL patients and healthy donors were collected from the biobanks of the Erasmus MC Hematology department (Rotterdam, The Netherlands), the University Hospital Regensburg Internal Medicine department (Regensburg, Germany) and the University Hospital Carl Gustav Carus (Dresden, Germany). Mononuclear cells were isolated from bone marrow or peripheral blood as described previously. Patients in the CIMP group were selected based on their high genome-wide methylation levels, whereas patients in other leukemia groups were selected to reflect the variety of mutational backgrounds in these disease. We are not aware of any (self select) biases that could affect the outcome of the study.
Ethics oversight	All patients provided written informed consent in accordance with the Declaration of Helsinki. The Medical Ethical Committee of the Erasmus MC has approved usage of the patient rest material for this study. The Medical Ethical Committee (Medisch Ethische ToetsingsCommissie Erasmus M, https://www.erasmusmc.nl/nl-nl/pages/metcC) of the Erasmus MC has approved usage of the patient rest material for this study.

Note that full information on the approval of the study protocol must also be provided in the manuscript.

Field-specific reporting

Please select the one below that is the best fit for your research. If you are not sure, read the appropriate sections before making your selection.

[x] Life sciences [ ] Behavioural & social sciences [ ] Ecological, evolutionary & environmental sciences

For a reference copy of the document with all sections, see nature.com/documents/nr-reporting-summary-flat.pdf

Life sciences study design

All studies must disclose on these points even when the disclosure is negative.

Sample size	We collected data from 376 leukemia patients and 19 donors in total, although not all data were available for every individual. All available CIMP patients (n=14) were used, having been previously identified on the basis of their methylation and gene expression profiles. For AML, a cohort representative of recurrent genetic abnormalities was used (n=224). RNA-seq was previously available from another study, but MCIP-seq, ChIP-seq, ATAC-seq and Hi-C data were generated ad hoc for a fraction of those patients. Similarly, RNA-seq of T-ALL patients (n=114) was available from another ongoing study, but it was supplemented with additional epigenomics data.<br><br>No sample size calculation was performed, as the sample size was determined by the availability of samples and/or data. However, sample sizes for all the main phenotypic groups studied here (CIMP, AML, T-ALL and CD34+) were large enough to robustly identify statistically significant differences.
Data exclusions	Low quality ATAC-seq and ChIP-seq datasets, as determined by standardized quality criteria (such as TSS enrichment or fragments of reads in peaks) were excluded from the analysis. In particular, ATAC-seq from AML patient UKR201 was removed from the clustering analysis, but not from other analyses.
Replication	No technical replicates were generated. Biological replicates were available in the form of multiple patients sharing the same phenotype. The reproducibility of the findings was assessed by incorporating orthogonal evidence. For example, publicly available MethylationEPIC array data from AMLs and T-ALLs was used to confirm that CIMP patients exhibit strong methylation signatures simular to those of ETP-ALL.
Randomization	Randomization was not applicable to the current study because CIMP leukemias were compared to those without this phenotype. Patients included in control groups (AML, T-ALL) were selected in such a way that they reflected the variety of mutational backgrounds in those leukemias. Donors for CD34+ cells were randomly selected.
Blinding	Blinding was not applicable to this study, as identification of differences between groups was an integral part of it.

Reporting for specific materials, systems and methods

We require information from authors about some types of materials, experimental systems and methods used in many studies. Here, indicate whether each material, system or method listed is relevant to your study. If you are not sure if a list item applies to your research, read the appropriate section before selecting a response.

Materials & experimental systems

n/a	Involved in the study
[ ]	[x] Antibodies
[x]	[ ] Eukaryotic cell lines
[x]	[ ] Palaeontology and archaeology
[x]	[ ] Animals and other organisms
[x]	[ ] Clinical data
[x]	[ ] Dual use research of concern
[x]	[ ] Plants

Methods

n/a	Involved in the study
[ ]	[x] ChIP-seq
[x]	[ ] Flow cytometry
[x]	[ ] MRI-based neuroimaging

Antibodies

Antibodies used	H3K27ac (Abcam):<br>Anti-histone H3 acetyl K27 rabbit antibody<br>Art. Nr: ab4729<br>Used: 1µg/µL (2.5 µg/IP)<br><br>CTCF (Cell Signaling):<br>Anti-CTCF rabbit antibody<br>Art. Nr: 2899S<br>Used: 4ug/µL<br><br>TCF7 (Cell Signaling)

	Anti TCF7 rabbit antibody<br>Art. Nr: 2203S<br>Used: 2ug<br><br>CEBPA (Santa Cruz Biotechnology):<br>Anti-CEBPA goat antibody<br>Art. Nr: SC9314<br>Used: 5ug<br><br>PU.1 (Cell Signaling):<br>Anti PU.1 rabbit antibody<br>Art. Nr: 2266S<br>Used: 0.4ug
Validation	Validation of the antibodies directed against H3K27ac and CTCF was done as part of the ChIP-seq data analysis. Aligned tracks exhibited good enrichment metrics, as measured by fragment of reads in peaks (FRIP) as well as by TSS enrichment. Furthermore, peaks were found at places that can be expected.<br>The manufacturer also conducted extensive validation of these antibodies, as can be seen on their official website:<br>H3K27ac: https://www.abcam.com/products/primary-antibodies/histone-h3-acetyl-k27-antibody-chip-grade-ab4729.html<br>CTCF: https://www.cellsignal.com/products/primary-antibodies/ctcf-antibody/2899

Plants

Seed stocks	*Report on the source of all seed stocks or other plant material used. If applicable, state the seed stock centre and catalogue number. If plant specimens were collected from the field, describe the collection location, date and sampling procedures.*
Novel plant genotypes	*Describe the methods by which all novel plant genotypes were produced. This includes those generated by transgenic approaches, gene editing, chemical/radiation-based mutagenesis and hybridization. For transgenic lines, describe the transformation method, the number of independent lines analyzed and the generation upon which experiments were performed. For gene-edited lines, describe the editor used, the endogenous sequence targeted for editing, the targeting guide RNA sequence (if applicable) and how the editor was applied.*
Authentication	*Describe any authentication procedures for each seed stock used or novel genotype generated. Describe any experiments used to assess the effect of a mutation and, where applicable, how potential secondary effects (e.g. second site T-DNA insertions, mosiacism, off-target gene editing) were examined.*

ChIP-seq

Data deposition

☒ Confirm that both raw and final processed data have been deposited in a public database such as GEO.

☒ Confirm that you have deposited or provided access to graph files (e.g. BED files) for the called peaks.

Data access links<br>*May remain private before publication.*	Raw data:<br>https://ega-archive.org/datasets/EGAD00001011059<br>https://ega-archive.org/datasets/EGAD00001011060<br><br>Processed data:<br>https://www.ebi.ac.uk/biostudies/arrayexpress/studies/E-MTAB-13117<br>https://www.ebi.ac.uk/biostudies/arrayexpress/studies/E-MTAB-13119<br>https://www.ebi.ac.uk/biostudies/arrayexpress/studies/E-MTAB-14060
Files in database submission	All raw sequencing data files are deposited in public databases and can be accessed using the numbers links provided in this form. Peak files (in .narrowPeak format) are also available at the same repository.
Genome browser session<br>(e.g. UCSC)	A genome browser session can be created and provided upon request.

Methodology

Replicates	We generated a single technical replicate ChIP-seq data for every single patient or donor. However, given that most of the analyses involved groups of patients exhibiting the same disease, multiple biological replicates were available for each of those phenotypes.
Sequencing depth	H3K27ac ChIP libraries were sequenced single-end with a read length of 51 bp. The average depth was 32,575,032 reads (SD = 17,383,019), excluding multi-mapping reads. No reads were removed after the alignment.<br><br>CTCF, CEBPA, PU.1 and TCF7 ChIP libraries were sequenced paired-end with a read length of 100 bp. Including multi-mapping reads, the average depth was:<br>- CTCF: 67,266,741 fragments (SD = 38,214,227)<br>- CEBPA: 46,274,836 fragments (SD=10,183,054)

- PU.1: 53,380,814 fragments (SD=12,766,212)
- TCF7: 49,484,901 fragments (SD=14,439,066)

No reads were removed after the alignment.

Antibodies	H3K27ac (Abcam) ab4729<br>CTCF (Cell Signaling) 2899S<br>TCF7 (Cell Signaling) 2203S<br>CEBPA (Santa Cruz Biotechnology) SC9314<br>PU.1 (Cell Signaling) 2266S
Peak calling parameters	Peaks were called using MACS2 callpeak v2.2.7.1, keeping all duplicates and using the human genome option. For single-end samples, BAM was specified as file type, whereas BAMPE was used for paired-end samples.
Data quality	ChIP-seq data quality was evaluated as per ENCODE standards (Landt S et al, Genome Res 2012). Namely, for each sample we considered the following parameters: total depth, number of peaks, fraction of reads in peaks (FRIP), normalized strand coefficient (NSC) and relative strand correlation (RSC). All the samples analyzed here complied with the ENCODE standards.
Software	ChIP-seq reads were aligned to the human reference genome build hg19 with either bowtie (v1.1.1) for single-end data or bowtie (v2.3.4.1) for paired-end data. Bigwig files were generated for visualization with deepTools bamCoverage 96 (v3.5.1) and the options --normalizeUsing RPKM --smoothLength 100 --binSize 20. Peak calling was performed with MACS2 using default settings. Quality metrics were computed with an in-house algorithm.
